# Identification of the nature of reading frame transitions observed in prokaryotic genomes

**DOI:** 10.1093/nar/gkt274

**Published:** 2013-05-06

**Authors:** Ivan Antonov, Arthur Coakley, John F. Atkins, Pavel V. Baranov, Mark Borodovsky

**Affiliations:** ^1^School of Computational Science and Engineering at Georgia Tech, Atlanta, GA 30332, USA, ^2^Department of Biochemistry, University College Cork, Ireland, ^3^Department of Biological and Medical Physics, Moscow Institute of Physics and Technology, Dolgoprudny, Moscow Region 141700, Russia, ^4^Center for Bioinformatics and Computational Genomics at Georgia Tech and ^5^Joint Georgia Tech and Emory Wallace H Coulter Department of Biomedical Engineering, Atlanta, GA 30332, USA

## Abstract

Our goal was to identify evolutionary conserved frame transitions in protein coding regions and to uncover an underlying functional role of these structural aberrations. We used the *ab initio* frameshift prediction program, GeneTack, to detect reading frame transitions in 206 991 genes (fs-genes) from 1106 complete prokaryotic genomes. We grouped 102 731 fs-genes into 19 430 clusters based on sequence similarity between protein products (fs-proteins) as well as conservation of predicted position of the frameshift and its direction. We identified 4010 pseudogene clusters and 146 clusters of fs-genes apparently using *recoding* (local deviation from using standard genetic code) due to possessing specific sequence motifs near frameshift positions. Particularly interesting was finding of a novel type of organization of the *dnaX* gene, where recoding is required for synthesis of the longer subunit, τ. We selected 20 clusters of predicted *recoding* candidates and designed a series of genetic constructs with a reporter gene or affinity tag whose expression would require a frameshift event. Expression of the constructs in *Escherichia coli* demonstrated enrichment of the set of candidates with sequences that trigger genuine programmed ribosomal frameshifting; we have experimentally confirmed four new families of programmed frameshifts.

## INTRODUCTION

Protein encoding imposes constraints on genomic sequence. Because the constraints are frame dependent it is possible to infer from a genomic sequence, which one out of six possible reading frames is likely to be translated (if any). Recently, we have developed a computational method, GeneTack, for identifying such infrequent locations where protein coding instantly transits from one frame to another without presence of stop and start codons (1). In the present work, we use comparative genomics to classify frame transitions predicted by the new method in prokaryotic genomes. This approach is conceptually similar to one recently used in a study of bacterial genes annotated in GenBank as genes with disrupted open reading frames (ORFs) (2).

There are several reasons, both technological and biological, to observe frame transitions in prokaryotic genomes. On the technological side, sequencing and assembly errors produce artifacts subsequently incorporated into databases. Biological reasons are indel mutations, conserved in evolution frame transitions involved in non-standard mechanisms of transcription or translation known as *recoding* (3–8), phase variation, etc.

Many indel mutations that may truncate and inactivate protein products would not affect the rest of the sequence, particularly the promoter region. Therefore, a mutated gene (a pseudogene) may still be transcribed with the RNA potentially carrying a regulatory role; thus, in a certain lineage the pseudogene sequence may evolve under purifying selection. Frame transitions also appear in genes that use phase variation, e.g. at a specific hypermutable location (9,10).

Identification of genes with *recoding* from genomic sequence alone is a challenging task (11–13). New *recoding* events may reveal novel DNA sequence patterns (stimulatory sequences) required for switching to a non-standard mechanism of gene expression. Understanding the ways stimulatory sequences work can shed light on yet unknown details of transcription and translation machinery. It may also provide new synthetic biology means for controlling gene expression. Therefore, this study has a particular emphasis on identification of novel *recoding* candidates.

*Recoding* can work through a range of RNA editing mechanisms [slippage (14,15), guided RNA editing (16,17), adenosine and cytosine deamination (18–20)]. On the other hand, RNA transcripts may be subjects for a variety of translational *recoding* mechanisms [ribosomal frameshifting, codon redefinition, translational bypass, StopGo (21)]. Here we concentrate only on the mechanisms related to transitions between reading frames: ribosomal frameshifting and transcriptional realignment; given that these mechanisms have functional roles, they are often described as programmed, e.g. Programmed Ribosomal Frameshifting (PRF) and Programmed Transcriptional Realignment (PTR) (2).

On PRF a ribosome changes the initial reading frame at a specific location in mRNA. Displacements of a ribosome by +1 and −1 nucleotide have been predominant while displacements by +2, −2 and even up to 50 nucleotides (commonly known as bypassing) have been documented (22–26). While PRF has been detected in many prokaryotic and eukaryotic species, it is especially prevalent in viruses (5,27). High-efficiency PRF is modulated by a range of stimulatory signals at the RNA level (28,29). Other signals can also affect readout of mRNA either through complementary mRNA:rRNA pairing (30,31) or via nascent peptide interaction with the peptide exit tunnel of the ribosome (32,33).

The PTR event [also termed transcriptional slippage (15), stuttering (34), molecular misreading (35) and reiterative transcription (36)] occurs when realignment of a growing RNA chain to the DNA template within the RNA polymerase ternary complex results in insertion or deletion of a single or multiple nucleotides relative to the DNA template (37,38). The indels usually occur in characteristic motifs such as homopolymeric runs of adenines or thymines.

In prokaryotes, the best known examples of genes with *recoding* are Insertion Sequence (IS) elements, as well as genes for Release Factor 2 (*prfB*) and DNA polymerase III (*dnaX*). Over 80% of eubacterial species use +1 PRF during *prfB* expression (11,39). Fewer examples of *recoding* were reported for *dnaX* (7,40) whose expression in diverse organisms has been less studied. Interestingly, though in different species, both PTR and PRF are known to be used in expressing *dnaX* (40), suggesting that PTR and PRF, at least in some locations, are interchangeable. IS elements, in particular members of IS3 family, use both PRF and PTR for expressing protein products (2). *Recoding* mechanisms are especially abundant among viruses (5,41,42). In this study, we sought to identify previously unknown cases of *recoding* that involve frame transitions. Our bioinformatic approach is independent from prior annotation; this approach yielded a number of new *recoding* candidates with some subjected to experimental tests.

## MATERIALS AND METHODS

### Translation of predicted fs-genes; BLASTp and Pfam validations

A frame transition predicted by GeneTack (1) indicates the presence of two overlapping protein coding ORFs that may constitute a single gene with a frameshift, an ‘fs-gene’. Frequently, these two ORFs are annotated in databases as separate genes. For further analysis, we combine the first ORF with the second ORF at the position of the predicted frameshift taking into account the predicted frameshift direction (+1 or −1). Translation of the ORF extended in this manner yields an ‘fs-protein’. Thus, each predicted frameshift makes an fs-gene and a fs-protein.

The GeneTack false discovery rate (FDR) determined earlier on a set of 17 prokaryotic genomes is ∼32% (1). Given the relatively high FDR, we used two complementary methods to confirm GeneTack predictions. We used BLASTp search to find a protein in the NCBI nr database whose alignment to the fs-protein (the query) had a score with E-value <10^−^^10^. Moreover, the sequence alignment to database protein had to cover at least a 100 AA fragment of the fs-protein containing the predicted frameshift position (Supplementary Figure S1A). If, on the other hand, the BLASTp search for an fs-protein query produced two sets of BLASTp hits disconnected at the frameshift position (Supplementary Figure S1B), this result was indicative of the presence of a pair of overlapping genes. Then, the predicted frameshift was characterized as an instance of frame transition between two adjacent genes. Still, given the possibilities of gene fusion and fission, some of BLASTp validated frameshifts may yet be false positives, e.g. a frameshift predicted between adjacent genes whose homologs are fused in another genome (43).

The frameshift validation could be more substantial were a search against the Pfam domain database produced an alignment to a Pfam domain (with E-value <10^−^^3^) covering the predicted frameshift position. Assuming that a conserved domain could not be divided between two fused genes, the Pfam confirmation would exclude artifacts related to gene fission and fusion.

### Ribosome-binding site of the downstream protein-coding region

A ribosome-binding site (RBS) motifs are not expected to be specifically associated with frameshifts caused by indel (pseudogenizaton) mutations, as well as by sequencing error. However, some programmed frameshifts have stimulatory sequences of the Shine-Dalgarno type located near the start of the protein-coding part of the downstream ORF. The gene prediction program GeneMarkS (44) provides parameters and initial gene predictions for GeneTack as well as computes an RBS score for the upstream region of each predicted gene; in our observations the RBS score values range between −11 and 8 (a larger score corresponds to a stronger RBS). Normally, in a location of given fs-gene, GeneMarkS predicts two separate genes. If the downstream ‘gene’ is not a real full-length gene, its ‘RBS score’ is expected to be low. Therefore, GeneTack filters out fs-genes if the RBS scores for downstream coding region is >2.2 (1). Still, in analysis of clusters of fs-genes, even slightly elevated RBS scores that appear consistently in the whole cluster could be indicators of false-positive predictions. For 10 434 frameshifts confirmed by both BLASTp and Pfam, an average value of the RBS scores of downstream coding regions was −1, while the average value of the RBS scores for downstream coding regions in all the remaining fs-genes was −0.14.

### Clustering

All 206 991 predicted fs-proteins (with or without BLASTp and Pfam confirmations) were grouped into clusters based on sequence similarity and conservation of frameshift position and directionality (+1 or −1). First, in the database of all fs-proteins ‘all-against-all’ BLASTp search was performed with a stringent E-value threshold 10^−^^50^ chosen to avoid inclusion of non-homologous proteins in the clusters that would facilitate detection of conserved DNA motifs related to programmed frameshifts.

Next, a graph was built with nodes representing 206 991 fs-proteins. Two nodes were connected by an edge if (i) positions of two frameshifts were inside the BLASTp pairwise alignment block (separated from the block border by at least 10AA); (ii) both frameshifts had the same direction (+1 or −1); and (iii) the distance between frameshift positions in the block was ≤50AA. This graph-connected components with two or more nodes were called clusters (GeneTack clusters).

The fs-genes that did not cluster were likely to be related to genes sequenced with errors, orphan pseudogenes, pairs of overlapping orphan genes or even orphan genes with programmed frameshifts.

Then we proceeded with classification of clusters of homologous fs-genes as (i) fs-genes with programmed frameshifts; (ii) pseudogenes or hypothetical pseudogenes; (iii) fs-genes with phase variation; (iv) fs-genes with translational coupling; as well as (v) fs-genes related to overlapping pairs of homologous genes (false-positive clusters).

### Functional characterization of the GeneTack clusters

In a given GeneTack cluster, we expected to see fs-proteins with similar function. If more than 50% of fs-proteins in a cluster contained the same Pfam domain, its name was assigned to the cluster. Clusters of multi-domain proteins received a ‘multi-function name’ with more frequent domains listed first.

If the majority of fs-proteins in a cluster did not have a match to a Pfam domain, the cluster did not receive a Pfam-derived name (just a cluster ID), unless a functional cluster name was derived by BLASTp ‘function transfer’ from hits to the NCBI nr database produced by the fs-proteins.

### Identification of clusters of fs-genes using non-standard mechanisms of transcription and translation

**Figure 1.**
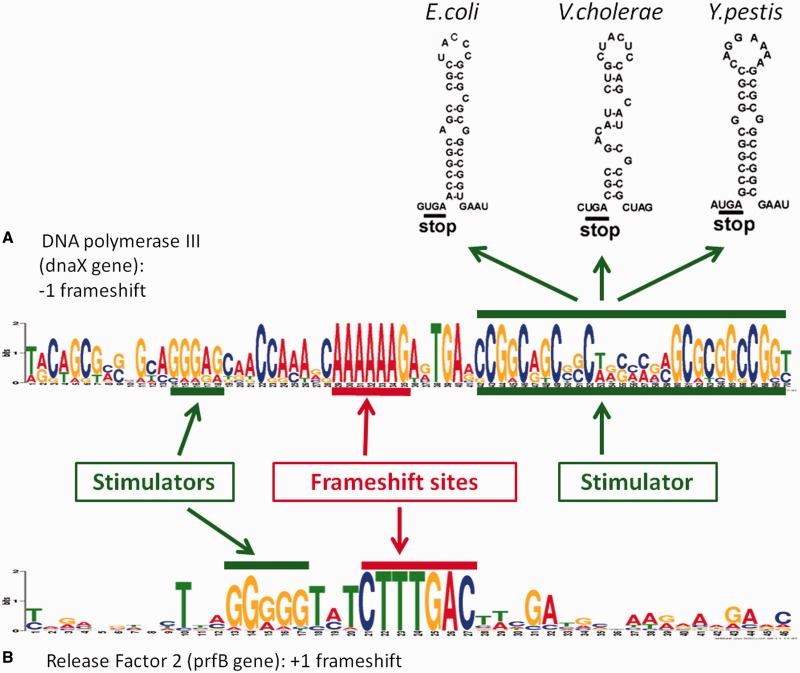
Examples of patterns facilitating programmed frameshifting. (**A**) ‘−1’ programmed frameshift is used in *dnaX* gene to express two subunits of DNA polymerase III. The Logo for (A) was derived from aligned sequences from 9 genera (*Escherichia*, *Salmonella*, *Neisseria*, *Vibrio*, *Shigella*, *Citrobacter*, *Enterobacter*, *Yersinia*, *Serratia*). The frameshift signal consists of conserved frameshift pattern AAA_AAA_G (‘slippery sequence’) and two stimulators. The upstream stimulator is a Shine-Dalgarno–like sequence that interacts with ribosome, while the downstream stimulator makes a hairpin secondary structure (7). (**B**) ‘+1’ programmed frameshift is used in *prfB* gene to auto regulate expression of Release Factor 2. The Logo for (B) was derived from 413 sequences (138 genera). The frameshift signal consists of conserved frameshift motif with consensus CTT_TGA_C and upstream Shine-Dalgarno–type sequence stimulator.

Transcriptional realignment and ribosomal frameshifting often occur at specific sequences where the PTR or PRF efficiency is augmented by additional *cis*-elements (Figure 1). Owing to the limited repertoire of shift- and slip-prone sequences, they evolve under purifying selection. In case of PTR, the specific sequences often appear to be homopolymers of eight nucleotides or longer, or combinations of two shorter homoploymers.

A PRF event involves rearrangements of two tRNAs interacting with two codons. For a +1 frameshift, the tRNA recognizing the A-site moves one nucleotide downstream, while in the case of a −1 frameshift, both tRNAs already occupying P- and A-site move one nucleotide upstream. Thus, in both cases, there are seven nucleotides involved in the PRF frameshift mechanism (45). On the other hand, the PTR slippage sites have been observed to be even longer (38). Therefore, it is expected that genuine instances of programmed frameshifting should be related to at least seven-nucleotide-long sequences evolving under purifying selection. Identification of conserved hepameric and longer sequences at the predicted frameshift site can be used as supportive evidence for programmed frameshifting.

**Figure 2.**
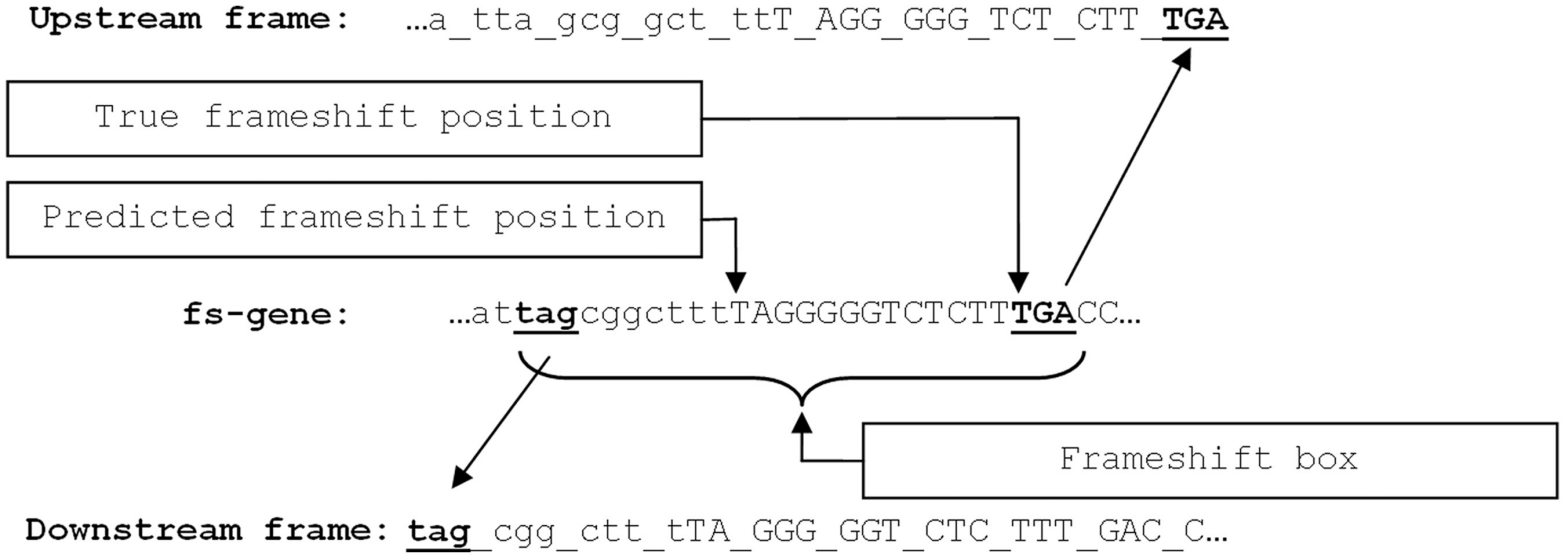
An example of a ‘frameshift box’. Predicted frameshift position appears in between two stop codons situated in different frames (TAG stop codon upstream and the TGA stop codon downstream). The true frameshift position is always located inside the ‘frameshift box’, the region between two stop codons.

To precisely delineate specific motifs related to PTR or PRF in a given cluster, we built a multiple alignment of ‘frameshift boxes’, sequences surrounding predicted frameshift positions. A frameshift box is bounded by two stop codons, one at the 5′-end of the downstream ORF and the other at the 3′-end of the upstream ORF (Figure 2). Both predicted and true frameshift positions should have occurred within the frameshift box. If the distance between the two stop codons was >100 nt (a frequent case in high GC genomes), the frameshift box was reduced to the 100-nt long vicinity of the predicted frameshift.

Several efficient algorithms and software tools for finding conserved motifs have been developed earlier [e.g. the Gibbs Sampler (GS) (46) and MEME (47)]. Still, in the case of PRF not only the motif *per se* but also the phase of motif with respect to reading frame, set by the start codon of upstream ORF, is important. For example, in the *prfB* gene encoding Release Factor 2, the consensus frameshift motif is YTT_TRA_C, with the triplet TRA being a stop codon. When phase is important (e.g. PRF motifs) the DNA sequence alphabet could be extended by an additional symbol indicating the frame of upstream ORF (an underscore symbol). Frameshift box sequences phased by underscores were used in a customized version of the GS algorithm to produce motifs with a given triplet phase. The consensus of motif sequences (a phased motif) was used to characterize the frameshift site in a given cluster.

To initially identify motifs prone to +1 and −1 programmed frameshifts, we searched for framed heptamers that occured in the frameshift boxes of a given cluster (N_NNN_NNN for −1 frameshifts and NNN_NNN_N for +1). Clusters containing between 5 and 100 fs-genes with average sequence identity of the frameshift box ≤80% were selected (1017 ‘−1’ clusters and 1380 ‘+1’ clusters). Starting positions of motifs were chosen randomly and GS was run 100 times searching for N_NNN_NNN motifs in −1 clusters and NNN_NNN_N motifs in +1 clusters.

For large clusters (with 100 or more fs-genes), alignments of the most over-represented heptamers were used to initiate the GS iterations. Consensus sequences of alignments found by the GS were recorded (framed heptamers). When motif positions for the first iteration were chosen randomly, consensus heptamers found in different GS run could vary. We recorded a number of times a heptamer *X* appeared as a GS consensus for a particular cluster. The score of a heptamer *X* was computed as follows:

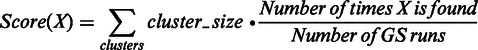

Notably, consensus heptamers containing a start codon for a downstream frame (AT_G for ‘+1’ and A_TG for ‘−1’) indicated that frameshifts were predicted at overlaps of pairs of homologous genes.

Among the consensus heptamers found in our analysis, there were seven A-rich heptamers (AAA_AAA_A, AAA_AAA_T, A_AAA_AAG, T_AAA_AAA, A_AAA_AAC, A_AAA_AAA and G_AAA_AAA) (Supplementary Table S1). It was reassuring to see this result, as it is well known that poly-A motifs frequently appear in frameshift sites.

To better identify possible stimulatory motifs, the alignments were extended 20 nt upstream and downstream from detected motifs of frameshift sites. We used positional nucleotide frequencies defined in extended alignments (with frame phase omitted) to build a logo (48) of sequence conservation at the frameshift site. Obviously, finding a conservation pattern did not guarantee that a given fs-gene cluster contained genes with programmed frameshifts. Evolutionary conserved sequences could be present at overlaps of homologous gene pairs. Therefore, we introduced several features for cluster classification as described in Table 1.

**Table 1.** Features (the first column) used to classify predicted frameshifts into types (the type names are given in the top two rows)

	Cluster type					Singleton type	
	Programmed frameshift	Phase Variation	Translational Coupling	Pseudogene	H-pseudogene	Pseudogene	H-pseudo / Error
Cluster contains 5 or more fs-genes	Yes	Yes	Yes	n/r	n/r	n/a	n/a
Conserved frameshift site	Yes	n/r	n/r	No	No	n/a	n/a
Cluster with small (≤2) number of genera	n/r	n/r	n/r	Yes	Yes	n/a	n/a
RefSeq annotation of a pseudogene^a^	n/r	n/r	n/r	Yes	No	Yes	No
Tandem repeat near frameshift position	n/r	Yes	n/r	n/r	n/r	n/r	n/r
ORF2 start is located close to ORF1 stop	n/r	n/r	Yes	n/r	n/r	n/r	n/r
BLASTp validation^b^	n/r	n/r	n/r	n/r	Yes	n/r	Yes
Pfam validation	n/r	n/r	n/r	n/r	n/r	n/r	Yes
Manual verification^c^	n/r	Yes	Yes	n/r	n/r	n/r	n/r

H-pseudo, hypothetical pseudogene; n/r, the feature is not required; n/a, the feature is not applicable; Error, sequencing error.

^a^A cluster must contain at least one annotated pseudogene.

^b^>50% of cluster fs-genes must be validated by BLASTp.

^c^Manual verification includes functional analysis of the fs-proteins and literature survey.

#### Inferring a type of frame transition mechanism

At poly-A/T sites, programmed frame transition may occur during either transcription or translation (for example in transposase and in *dnaX* genes). Sequence conservation features allow for selecting a specific hypothesis on the mechanism of programmed frameshift.

**Figure 3.**
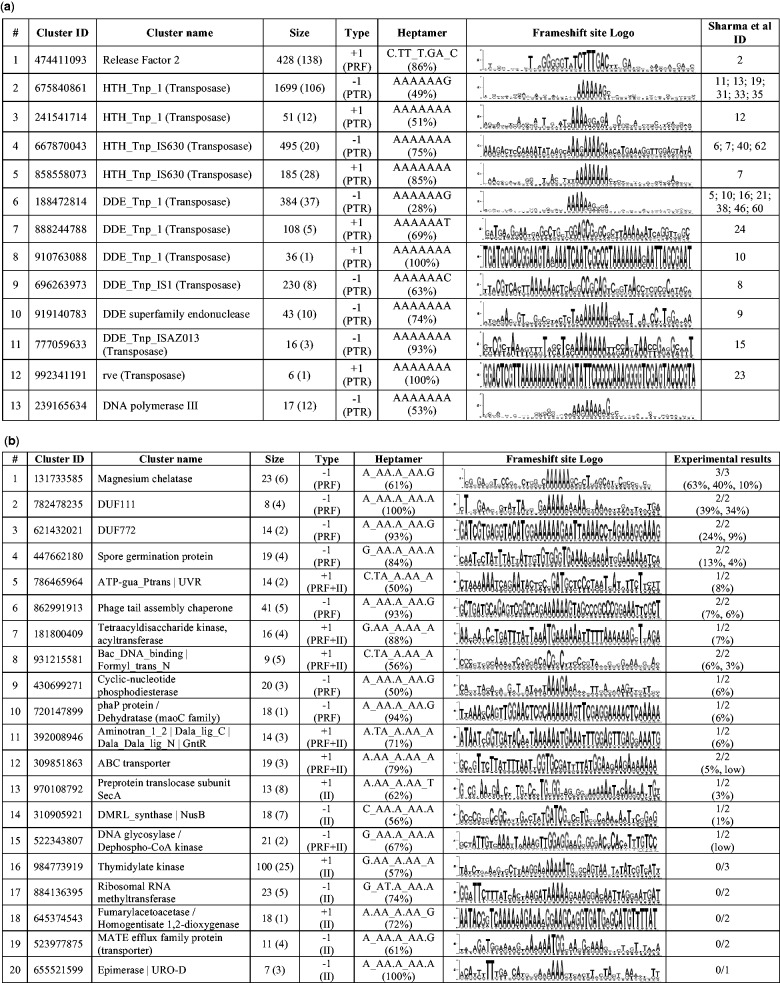
(**a**) List of GeneTack clusters corresponding to known cases of programmed frameshifting. #, row index; Cluster ID, unique identifier of a cluster (can be used for a search in GeneTack database); Cluster name, designated protein function; Size, number of fs-genes in the cluster (number of different genera is specified in parenthesis); Type, frameshift direction (possible mechanism: PTR, programmed transcriptional realignment, PRF, programmed ribosomal frameshifting, II, internal initiaion); Heptamer, overrepresented heptamer (the fraction of the cluster’s fs-genes that contain the heptamer); Frameshift site Logo, logo of the frameshift site (see text for details); Sharma *et al.* ID, ID of the corresponding Sharma et al cluster(s) (2). (**b**) Summary of predicted programmed frameshifts, selected from GeneTack clusters for experimental verification. First seven column headers are the same as in Figure 3a. Experimental results (X/Y)—X programmed frameshift candidates out of selected Y candidates from a given cluster have shown detectable level of frameshifting; numbers in parentheses give frameshifting efficiency (in percentage points) for the X candidates.

We have observed that in genes using PRF, the frameshift direction, +1 or −1, is conserved among orthologs (for example, all *prfB* genes use +1 shifts). On the other hand, our data showed that in genes with confimed PTR, such as transposase genes, the orthologs do not necessarily keep the same frameshift direction. Thus, for a single transposase family, our clustering approach produced two separate clusters, with +1 and −1 frameshifts. For example, the HTH_Tnp_IS630 family had 495 members in the ‘−1’ cluster and 185 members in the ‘+1’ cluster (Figure 3). In each cluster, only a framshift in specific direction could lead to synthesis of a full-length transposase.

Given these observations, if there were two or more clusters of fs-genes with the same function but different frameshift direction, the predicted nature of frameshift was PTR; otherwise, if only one direction of frameshift was ubiquitous in a cluster, the predicted mechanism was PRF.

Interestingly, in some experimentally confirmed genes with *recoding*, the mechanism of programmed frameshifting is still under debate as even orthologous genes may use different *recoding* mechansms. For example, expression of *dnaX* genes goes via PRF in some prokaryotic species and via PTR in other (40,49).

### Frame transitions related to phase variation and translational coupling

*Phase variation*, a reversible and inheritable change of bacterial phenotype is often considered as a random process evolved to facilitate evasion of a host immune respond. Among molecular mechanisms of phase variation (homologous recombination, inversion of DNA elements, etc.), slipped strand mispairing (SSM) seems to be the most common. During replication, SSM may occur at repeat units (such as short sequence repeats, microsatellites or variable-number tandem repeats). The repeat unit could be as simple as a homopolymer sequence [e.g. poly-A in the *p78* gene of *Mycoplasma fermentans* (50) or poly-C/poly-G in the type III methyltransferases genes (51)] or a repeat of more complex subunits (for example AGTC is repeated >30 times in the *mod* gene of *Haemophilus influenzae*). Insertion or deletion of a repeat unit on replication creates a frameshift mutation to turn the gene on and off.

Phase variation has been studied mainly in bacterial pathogens; however, it may occur in non-pathogens as well (52). The majority of proteins encoded by genes involved in phase variation are exposed to the cell environment. Examples include proteins involved in capsule, fimbriae, pili, flagella as well as surface proteins: transporters, receptors and porins. Notably, many of large GeneTack clusters contain fs-genes for cell surface and secretory proteins. Still, phase variation has also been associated with DNA modification and metabolism-associated genes (53).

As poly-AT is a slippery sequence for DNA polymerase (as well as for RNA polymerase and ribosomes), a stretch of poly-AT could cause phase variation. For a given cluster, we computed a fraction of fs-genes with a poly-AT stretch (minimal length 7 nt) close to the frameshift, designated as %AT. Finally, we used the tandem repeat finder program (54) to identify other types of repeats (such as poly-G or poly-GC). The program parameters were set to report homopolymers as repeats (minimal length 7 nt). For a given cluster, we determined a fraction of fs-genes with tandem repeats (other than Poly-A and poly-T) near a predicted frameshift, designated as %R.

Clusters of fs-genes were classified as related to phase variation if (i) we observed characteristic repeats near the frameshift position as well as (ii) function of some fs-genes in the cluster was earlier associated with phase variation.

*Translational coupling* of two adjacent genes implies existence of a re-initiation mechanism that requires proximity of the translation initiation site of downstream gene to the translation termination site of upstream gene. The downstream gene may have a weak RBS site. Gene pairs with evolutionary conserved translational coupling could be predicted as fs-genes and could form clusters. Observation of evolutionary conservation of co-location of upstream ORF stop and downstream ORF start codons would support a translational coupling hypothesis. For a cluster with ≥100 fs-genes, we determined a fraction (%S) of fs-genes with co-localized starts and stops (within 10 nt distance). A high value of %S was considered to be a signature of translational coupling. Phase variation or translational coupling characterization, as shown in Table 2, was based on the combination %S, %R and %AT values.

**Table 2.** The largest clusters containing 100 or more fs-genes

Cluster ID	Cluster name	Size	#G	D	%AT	%R	%S	%B	BR
474411093	Release factor 2	428	138	+1	49	4	1	2	PF^a^
675840861	HTH_Tnp_1 (Transposase)	1699	106	−1	72	7	3	75	PF
188472814	DDE_Tnp_1 (Transposase)	384	37	−1	67	4	2	85	PF
888244788	DDE_Tnp_1 (Transposase)	108	5	+1	80	0	0	95	PF
667870043	HTH_Tnp_IS630 (Transposase)	495	20	−1	98	1	0	96	PF
858558073	HTH_Tnp_IS630 (Transposase)	185	28	+1	100	5	0	86	PF
696263973	DDE_Tnp_IS1 (Transposase)	230	8	−1	90	0	0	72	PF
784826247	Transposase IS911/IS222	112	5	−1	6	0	0	0	PF
752989859	Kinase/Phosphatase	105	23	+1	67	1	16	0	PF
279791230	HATPase_c, HisKA, Response_reg	594	148	+1	57	5	35	13	PV
487884579	HATPase_c, HisKA, Response_reg	292	98	+1	36	18	31	35	PV
107592512	HATPase_c, HisKA, Response_reg	162	51	−1	36	4	56	5	PV
437298609	BPD transporter	238	79	+1	34	9	34	5	PV
672517721	BPD transporter	149	46	+1	41	14	42	26	PV
953823467	BPD transporter	100	22	−1	5	17	10	0	PV
6376240	tRNA synthetase	215	81	+1	60	7	36	1	PV
138502135	Aminotransferase	175	88	+1	38	13	30	13	PV
354349696	Secretion system	140	51	+1	41	6	18	9	PV
322052632	Fucose synthase / Dehydratase	139	78	+1	44	9	37	4	PV
631171255	PqiA integral membrane protein	126	38	+1	71	4	17	5	PV
222950006	ABC transporter	436	116	+1	46	7	47	59	TC
785097185	ABC transporter	298	66	+1	62	4	48	63	TC
208900412	ABC transporter	293	97	+1	24	4	61	69	TC
624178257	ABC transporter	289	102	−1	49	8	45	64	TC
79330857	ABC transporter	280	97	+1	35	9	86	1	TC
104388297	ABC transporter	146	61	−1	21	18	64	11	TC
22890314	ABC transporter	144	49	+1	24	3	65	69	TC
471276212	ABC transporter	126	48	+1	25	2	60	33	TC
548076848	Flagella	139	34	+1	48	3	71	0	TC
585180489	Flagella	111	36	+1	8	11	75	0	TC
181132644	Flagella	118	46	+1	36	10	70^2^	0	TC
847934252	Polyketide cyclase	132	38	+1	46	4	81^2^	0	TC
697472870	Biotin carboxylase	128	44	+1	73	5	75	2	TC
876288400	Hydrolase/Epimerase	121	27	+1	28	2	79	0	TC
458305551	Polyphosphate kinase	113	35	+1	27	8	63	2	TC
237996460	Mur ligase	112	33	+1	78	3	83^1,2^	90	TC
717516549	Epimerase	111	65	−1	44	13	61	2	TC
539781944	Oxidoreductase	109	51	−1	39	1	80	0	TC
515287573	Recombination factor RarA	104	52	+1	55	5	74^2^	4	TC
984773919	Thymidylate kinase	100	25	+1	93	7	3	3	TC*

Size, number of fs-genes in the cluster; #G, number of different genera in the cluster; D, frameshift direction; %AT, fraction of fs-genes with 7+ nt poly-AT stretch located near predicted frameshift; %R, fraction of fs-genes with tandem repeats located near predicted frameshifts; %S, fraction of fs-genes with ORF2 start codon ATG (^1^GTG) located within 10 nt (^2^20 nt) from the ORF1 stop codon; %B, fraction of fs-proteins validated by BLASTp against NCBI nr database; BR, predicted biological role (PF, programmed frameshifting, PV, phase variation, TC, translational coupling).

^a^Experimentally verified.

### Experimental verification of predicted programmed frameshifting

#### Bacterial strains

The *E. coli* strains DH5α and MG1655Δ*lacIZ* were used for plasmid propagation and western blot analyses, respectively. Strains were grown in Luria–Bertani (LB) plus or minus isopropyl-β,d-thiogalactopyranoside (IPTG).

#### Plasmid construction

The vector pJ307 was derived from the GST-MBP-His fusion vector (pGMH57) by ligating annealed oligonucleotides (5′-GATCAGCTCGAGCACTAGTCCATGGGGATCCAAG-3′ and 5′-AATTCTTGGATCCCCATGGACTAGTGCTCGAGCT-3′) into pGMH57 between BamHI-EcoRI restriction sites of pGHM57 (55). Twenty inserts were constructed by PCR amplification of complementary oligonucleotides to produce a full-length sequence containing 5′ XhoI and 3′ BglII restriction sites. These fragments were restriction digested and then ligated into the vector pJ307, digested by compatible restriction enzymes PspXI and BamHI (present in the new cloning site of pJ307), so that the *MBP* gene was in an alternative frame (+1 or −1) relative to *GST* or in-frame for positive control. Supplementary Table S2 shows the full-length sequences of the inserts.

#### Western blot analysis

Overnight cultures of strains expressing the appropriate plasmid were diluted 1:100 in LB Broth, grown for 2 h at 37°C, and then induced with 100 mM IPTG for an additional 2 h at 37°C. Crude extracts were obtained by culture centrifugation and re-suspending the bacterial pellet in Laemmli sample buffer. Proteins were separated on 10% sodium dodecyl sulphate polyacrylamide gel electrophoresis gels and transferred to nitrocellulose membranes (Protran). Immunoblots were incubated at 4°C overnight in 5% milk/phosphate buffered saline–Tween containing a 1:500 dilution of rabbit anti-GST or 1:2000 dilution of rabbit anti-HIS. Immunoreactive bands were detected on membranes after incubation with appropriate fluorescently labeled secondary antibodies using a LI-COR Odyssey® Infrared Imaging Scanner (LI-COR Biosciences). The amounts of termination and frameshift product were quantified by ImageQuant. The frameshifting efficiency was estimated as the ratio of the amount of frameshift product to the total amount of termination plus frameshift products.

## RESULTS

### Frame transitions predicted in 1106 genomes

We downloaded 1106 prokaryotic genomes longer than 1 Mb (77 archaeal and 1029 bacterial; see Table 3 for information on phylogenetic diversity) from the NCBI Web site ftp://ftp.ncbi.nih.gov/genomes/Bacteria/all.gbk.tar.gz (on 12 April 2010; draft genomes were excluded). The GeneTack-GM software program (1) with default settings was used to screen all the sequences; 206 991 frameshifts were predicted. The number of predicted frameshifts in a given genome has shown correlation with its length and the number of genes (see Supplementary Figure S2). As the GeneTack accuracy in frameshift detection is characterized by 32.8% FDR, we expected about one-third of the predictions to be related to frame transition between adjacent genes rather than to frameshifts. For translations of 36 668 (17.7%) fs-genes, BLASTp detected the NCBI nr database homologous proteins ‘bridging the frameshifts’; also the Pfam domains covering predicted frameshifts were detected for 16 307 fs-genes (both continuous BLASTp hits and Pfam domains existed for 10 434 fs-genes). We have observed that 18 436 predicted fs-proteins resulted in ‘split BLASTp hits’ indicative of false-positive prediction. All 206 991 fs-genes and fs-proteins were used in the analysis described below.

**Table 3.** Phylogenetic distribution of the species and genomes analyzed by GeneTack

	Taxon	Number of species	Number of genomes	Number of fs-genes	Number of fs-genes/Mb	% fs-genes in clusters
Bacteria	*Acidobacteria*	3	3	1201	60.8	9%
	*Actinobacteria*	72	91	24 494	58.4	29%
	*Aquificae*	7	7	1238	105.2	39%
	*Bacteroidetes*	20	22	4910	50.8	34%
	*Chlamydiae*	7	15	1720	94.8	84%
	*Chlorobi*	10	11	2118	73.5	47%
	*Chloroflexi*	10	14	2265	49.2	47%
	*Cyanobacteria*	14	38	8425	64.8	49%
	*Deferribacteres*	2	2	460	84.3	41%
	*Deinococcus-Thermus*	5	6	1179	79.8	36%
	*Dictyoglomi*	2	2	327	85.7	52%
	*Elusimicrobia*	2	2	309	111.6	16%
	*Fibrobacteres*	1	1	130	33.8	18%
	*Firmicutes*	105	199	25 890	42.4	55%
	*Fusobacteria*	4	4	468	43.7	19%
	*Gemmatimonadetes*	1	1	453	97.7	25%
	*Nitrospirae*	1	1	240	119.8	35%
	*Planctomycetes*	2	2	504	37.8	18%
	*Proteobacteria*	315	574	110 466	53.9	55%
	*Spirochaetes*	6	10	2288	73.5	77%
	*Synergistetes*	2	2	218	56.9	32%
	*Tenericutes*	5	5	436	71.7	19%
	*Thermobaculum*	1	2	158	50.9	25%
	*Thermotogae*	11	11	1350	62.1	48%
	*Verrucomicrobia*	4	4	690	47.1	24%
Archaea	*Crenarchaeota*	17	23	7390	146.8	60%
	*Euryarchaeota*	49	52	7351	59.7	27%
	*Korarchaeota*	1	1	174	109.4	19%
	*Thaumarchaeota*	1	1	139	84.5	27%
Total		680	1106	206 991		

The number of genomes for a given species depends on the number of sequenced strains.

### About 50% of fs-genes were clustered

The clustering procedure described in ‘Materials and Methods’ section grouped 102 731 fs-genes into 19 430 clusters. The majority of the clusters contained a small number of fs-genes: 48% contained only two fs-genes and ∼75% of clusters contained less than five fs-genes. The abundance of small clusters was a result of using the stringent BLASTp threshold. Some small clusters could be related to fission events in a lineage (56).

Notably, a few clusters with up to several dozen fs-genes had similar or even identical sequences originated from closely related genomes such as genomes of 30 *E. coli* strains. Some fs-genes were detected in several copies in the same genome (e.g. genes for transposases).

### Predicted programmed frameshift clusters

A cluster of fs-genes with conserved motifs located uniformly close to predicted frameshift positions was characterized as a programmed frameshift cluster. We used the GS method (see ‘Materials and Methods’ section) to align frameshift box sequences and identify conserved motifs. This approach, as expected, detected several known families of genes with programmed frameshifts; corresponding conserved motifs were identified.

Many known ‘slippery’ sequences include poly-A/T stretches [such as A_AAA_AAG (57,58) and A_AAA_AAA (59) implicated in PRF or A_n_, n > 7 (14,40,60) and T_n_, n > 8 (61) involved in PTR). Poly-A/T sequences are prone to frameshifting during translation, transcription or even replication [as DNA polymerase may produce indel errors at poly-A/T stretches (62)].

Among clusters containing at least five fs-genes, we found 146 where at least 50% of the fs-genes contained one of the seven heptamers mentioned in ‘Materials and Methods’ section. These clusters (with 4302 fs-genes) were divided into two groups: (i) clusters of fs-genes with known programmed frameshifts (Figure 3a) and (ii) new clusters of fs-genes predicted to use programmed frameshifts (Figure 3b).

#### Fs-genes with known programmed frameshifts

The Recode-2 database contains a comprehensive collection of confirmed *recoding* events, (mainly of PRF type) in prokaryotes, eukaryotes and viruses, nearly ∼1500 entries (63). The recent work by Sharma et al. (2) extended the collection of known programmed frameshifts. First, Sharma et al. (2) conducted ‘all against all’ searches among conceptually translated protein products of disrupted coding regions annotated in GenBank. Second, the protein products were grouped into clusters of orthologs; however, the clustering did not take into account frameshift position and direction. Additionally, tBLASTn searches against the NCBI nr database were used to enrich the clusters with orthologous sequences not annotated as disrupted protein-coding regions. This approach produced 49 clusters with 8032 fs-genes.

To establish correspondence between clusters identified by Sharma et al. and 146 GeneTack clusters, each of the 8032 fs-genes was used as a query in a BLASTn search against 4302 fs-genes in GeneTack clusters.

We identified 12 GeneTack clusters with fs-genes having significant sequence similarity to the fs-genes in 26 clusters of Sharma et al. We provide information on these 12 clusters in Figure 3a (11 clusters of transposase genes and a cluster of *prfB* genes).

#### Transposase fs-genes

In our data, genes of transposases constitute the largest group of genes with known programmed frameshifts. Interestingly, in the family of DDE_Tnp_1 transposases, we identified six clusters (three with +1 and three with −1 shift direction). Only three of them (the largest in size) matched corresponding Sharma et al. clusters. Other two clusters with −1 frameshift (of size 6 and 29) and one with +1 frameshift (6 fs-genes) could present new branches in the transposase family. In total, we identified 7 new clusters of transposase fs-genes with size ranging from 5 to 29 fs-genes. Presence of A-rich sequences in frameshift sites and existence of +1/−1 cluster pairs in a single transposase family suggest that transposase fs-genes are likely to use the PTR mechanism (see Figure 3a).

#### prfB fs-genes

GeneTack detected frameshifts in 428 *prfB* genes encoding Release Factor 2. Expression of *prfB* uses PRF to produce full-length Release Factor 2 if its cell concentration becomes too low; regulation of *prfB* is one of the best-studied PRF instances (64). The *prfB* genes were grouped in a single cluster. The cluster could be even larger in size given that ∼70% of all eubacteria are expected to use PRF to regulate expression of *prfB* (39). Still, some *prfB* genes escaped frameshift detection such as genes with frameshifts located closer than 50 nt to the start codon.

#### dnaX fs-genes

We have discovered a new structural type of *dnaX* genes. The GeneTack *dnaX* cluster contains 17 novel fs-genes (from 12 genera) distinctly different from seven genes (four genera) present in the Recode-2 database. None of the predicted 17 *dnaX* fs-genes with programmed frameshifts were annotated in RefSeq. These 17 *dnaX* fs-genes contain −1 frameshifts at the DNA level.

Notably, use of programmed frameshifts in the family of *dnaX* genes encoding τ and γ subunits of DNA polymerase III is well-known. The current version of the Recode-2 database (63) contains *dnaX* genes annotated as a single ORF in genomes of *Escherichia*, *Neisseria*, *Salmonella* and *Vibrio* genera. The τ subunit is the full-length product of *E. coli dnaX* gene, while the γ subunit is the shorter −1 frameshift-derived product; the N terminal regions of τ and γ subunits are identical (65–67). The *E. coli dnaX* gene was proved to use the PRF mechanism triggered by sequence A_AAA_AAG (49). Interestingly, PTR at a stretch of 9 As was shown to be used to produce γ subunit in *Thermus thermophilus* (40). Effectiveness of the *dnaX* frameshifting was shown to be 50%, which is in line with the DNA polymerase III complex stoichiometry (68).

In the *dnaX* cluster, whose 17 fs-genes span two adjacent ORFs, 12 fs-genes have poly-A stretches: nine have 10 As (see Supplementary Figure S3) and the three remaining (not shown in Supplementary Figure S3) have nine As (*Chloroherpeton thalassium*), eight As (*Chlorobium chlorochromatii*) and seven As (*Chlorobium phaeobacteroides*). The sequences with 10As can be well aligned with the poly-A motif of *T. thermophilus,* thus suggesting the same PTR mechanism for the nine species with 10As. Arguably, the *dnaX* genes with shorter poly-A motifs use PTR as well.

This type of *dnaX* genes containing two ORFs has not been described earlier.

#### Experimental confirmation of predicted programmed frameshifting

The remaining 134 GeneTack clusters may also contain new genes with programmed frameshifts. To experimentally verify predicted programmed frameshifts, we manually selected 40 fs-genes from 20 clusters (out of the 134 clusters) that had the most pronounced conservation around the predicted frameshift site (see Supplementary Table S2). Putative frameshift-containing sequences were cloned in vector pJ307 (see ‘Materials and Methods’ section). This vector has a strong promoter, pTAC, with a lac operator, the glutathione S-transferase (GST) gene lacking a terminator and fused in-frame to a maltose-binding protein (MBP) gene with a PSPXI-BamH1 cloning site between GST and MBP. The plasmid separately encodes the LacIq repressor so that expression from the pTAC promoter is inducible by addition of IPTG. The cassettes of putative frameshift-relevant sequences were inserted at the cloning site and framed to yield the fusion protein on the frameshifting in the predicted direction; the frequency of observation of the termination product synthesized without framehifting characterizes the frequency of events when ribosomes fail to change frame. The frameshift efficiency was defined as the ratio of frameshift-derived product vs total of frameshift- and non-frameshift–derived products (Supplementary Figure S4). In another experiment, we measured translational coupling (internal initiation). This test involved a His-tag encoding sequence at the 3′ end of the MBP gene with quantification by western blots with His-tag–specific antibody. The results (Supplementary Figure S5) complemented those with anti-GST western blots for frameshift identification.

We observed >10% frameshifting efficiency on testing predicted *recoding* fs-genes from four clusters: magnesium chelatase (frameshifting efficiency up to 63%), DUF111 (up to 39%), DUF772 (up to 24%) and Spore germination protein (up to 13%).

**Figure 4.**
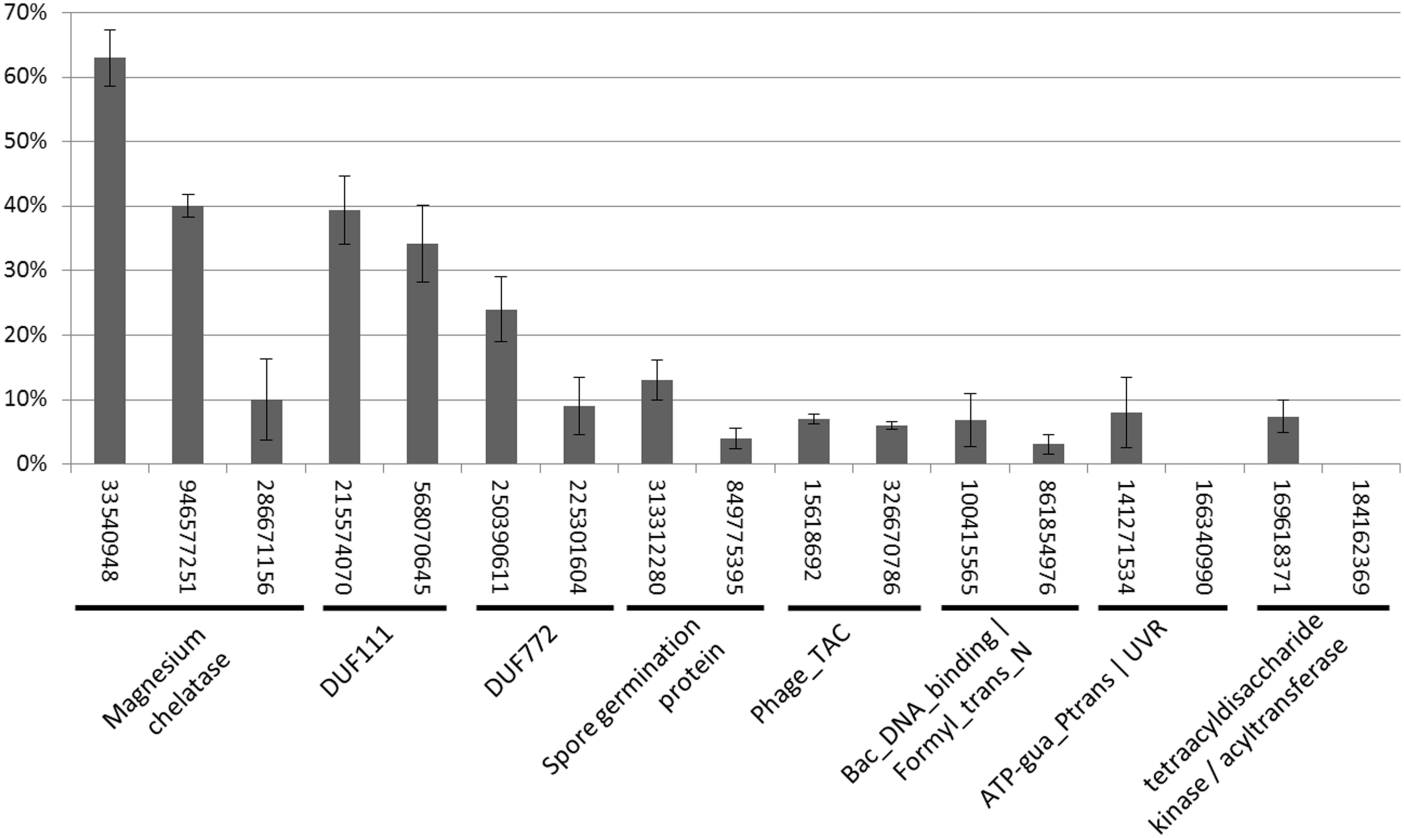
Experimental validation of predicted programmed frameshifting. The frameshifting efficiency in each experiment was estimated as the ratio of the product translated with the frameshift to the total amount of products translated with and without frameshift. The fs-gene ID’s are listed below the graph along with the names of clusters. Note that in the last two clusters, frameshifting was observed for only one of the constructs.

Genes for magnesium chelatase make a cluster of 23 fs-genes (with a −1 frameshift) from six different genera [in both the bacterial domain (*Pseudomonas*, *Burkholderia*, *Delftia* and *Herpetosiphon*) and the archaeal domain (*Methanocaldococcus* and *Methanococcus*)]. Cassettes made from three of the fs-genes were tested and significant levels of frameshifting, 63, 40 and 10%, were observed (Figure 4). Interestingly, the lengths of poly-A runs in the cassettes correlate with the frameshift efficiency (62): A_AAA_AAA_AAA_A (63%—11As), A_AAA_AAA_AA (40%—9As) and A_AAA_AAA (10%—7As) (Supplementary Table S2).

The newly predicted magnesium chelatase fs-genes with programmed frameshifts were annotated in RefSeq as two adjacent genes (each ∼1000 nt long) with a gene for magnesium chelatase annotated upstream and some other gene annotated downstream in the gene pair. Notably, a BLASTp search against NCBI nr database reveals several magnesium chelatase proteins (from *Chloroflexus aggregans*, *Rubrobacter xylanophilus* and others) made by a fusion of the two parts, an indication that the fusion protein and the proteins whose synthesis requires a *recoding* event are likely to have similar function.

Clusters DUF111 and DUF772 were named with respect to the Pfam domains of ‘unknown’ type detected in these fs-proteins (with DUF standing for ‘Domain of Unknown Function’). Genomic regions containing fs-genes from the DUF111 cluster are annotated in RefSeq as carrying two hypothetical genes, while in place of fs-genes from the DUF772 cluster annotation shows two separate transposases genes (IS1182 family). For fs-proteins from each cluster, similarly to the magnesium chelatase case, BLASTp searches against the NCBI nr database yield hits to fusions of two annotated proteins.

Interestingly, although the fs-genes from the Spore germination protein cluster are annotated as two separate genes encoding ‘Spore germination protein’, BLASTp did not produce fusion protein hits in the NCBI nr database. Still, in experiments we observed that a programmed frameshifting results in the fusion product.

We observed frameshifting efficiency of <10% in fs-genes from three clusters: phage tail assembly chaperone (7%), cyclic-nucleotide phosphodiesterase (6%) and phaP protein/dehydratase (6%). The fs-proteins from the phage tail assembly chaperone cluster (41 fs-genes) have significant similarity to a protein from *Enterobacteria phage HK97*; fs-genes encoding these fs-proteins have phage origin and are likely to use *recoding* (41,42). Still, in experiments with two fs-genes from this cluster we observed frameshifting efficiencies of 6% and 7%.

The fs-proteins from cyclic-nucleotide phosphodiesterase cluster have hits to fused proteins in the NCBI nr database suggesting similar function for products of the *recoding* fs-genes and fused genes, akin to the case of magnesium chelatase family.

Notably, a potential limitation of our experimental analysis is that frameshifting in sequences originated from different bacteria and even archaea was tested in *E. coli*. It is known that many frameshifting cassettes do not work in cross-species conditions. Thus, what we have observed, in attempt to assess the efficiency of genuine frameshiftings, is likely to give us an underestimate of the true efficiency. The fact that only parts of the genes were inserted between the reporters also contributes to producing false-negative observations. It is possible that we saw no frameshift in a particular case because the inserted sequence was too short to carry a crucial stimulatory signal. Examples of distant modulators of ribosome frameshifting are known. In *Saccharomyces cerevisiae* Antizyme mRNA modulator sequences are located at the ends of coding region (69). In Barley yellow dwarf virus a stimulatory signal was identified ∼4000 nucleotides downstream of the frameshift site (70). These considerations are especially relevant for the experiments produced neither frameshifting nor initiation.

### Other large clusters of fs-genes

#### Phase variation clusters

To identify putative phase variation clusters, we have taken the following approach. We collected a set of 38 genes with phase variation caused by the slipped strand mispairing (SSM) mechanism (53) (Supplementary Table S3). We used protein products of these genes in BLASTp searches (with E-value 10^−^^10^) against the database of all fs-proteins. For the 14 queries we observed hits to 13 clusters with five or more members (Supplementary Table S4). These 13 clusters were likely to contain fs-genes with conserved phase variation. Next, we attempted to detect poly-AT stretches and short tandem repeats characteristic for phase variation (see ‘Materials and Methods’ section) in the 50 nt vicinity of predicted frameshifts; the %AT and %R values were determined for each cluster (Table 2). A cluster was classified as a phase variation cluster if %AT and/or %R were higher than %S, an indicator for translational coupling (see ‘Materials and Methods’ section).

#### Translational coupling clusters

We observed that 137 clusters with five or more members contained genes for ABC transporters (4560 fs-genes), with eight clusters containing >100 members (Table 2). Earlier, it was experimentally shown that genes of ABC transporters use translational coupling, e.g. *drrAB* genes from *Streptomyces peucetius* (71), which protein products have shown similarity to fs-proteins from a GeneTack ABC transporter cluster (with 36 fs-genes). We characterized the nature of frame transitions in the ABC transporter clusters as translational coupling (Table 2).

Interestingly, the *p78* gene from the ABC transporter operon in *Mycoplasma fermentans* was characterized in (50) as a gene undergoing phase variation. However, the protein product of this *p78* gene did not have a match in our data.

Although we did not observe frameshift-derived products for several constructs used in our experiments, we did observe in such cases initiations of translation resulting in synthesis of a downstream gene products labeled with His-tag. Such observations are likely to confirm instances of translational coupling suggesting, given its conservation in fs-gene clusters, that such a co-regulation contributes to organism fitness. The clusters of fs-genes classified as translational coupling include Thymidylate kinase, Ribosomal RNA methyltransferase, Fumarylacetoacetase/Homogentisate 1,2-dioxygenase, MATE efflux family protein (transporter) and Epimerase /URO-D (Figure 3b).

Interestingly, according to experimental data, both programmed frameshifts and translation coupling may occur in fs-genes of seven clusters: ‘DNA glycosylase / Dephospho-CoA kinase’, ‘Bac_DNA_binding/Formyl_trans_N', ‘ABC transporter’, ‘DMRL_synthase / NusB’, ‘Preprotein translocase subunit SecA’, ‘Aminotran_1_2 / Dala_Dala_lig_C / Dala_Dala_lig N / GntR’, ‘ATP-gua_Ptrans / UVR’ and ‘Tetraacyldisaccharide kinase /acyltransferase’ (Supplementary Figure S5).

### Pseudogene clusters

There were 59 318 pseudogenes annotated in 1106 genomes: notably no single pseudogene was annotated in 265 genomes, while over a thousand pseudogenes were annotated in several genomes; e.g. in the parasitic bacteria *Mycobacterium leprae* (NC_011896), 1116 genes out of 2770 were annotated as pseudogenes. Notwithstanding the variability of the number of pseudogenes per genome depending on evolutionary path of a species, a low number of annotated pseudogenes in a genome could be related to far from perfect methods of pseudogene annotation.

**Figure 5.**
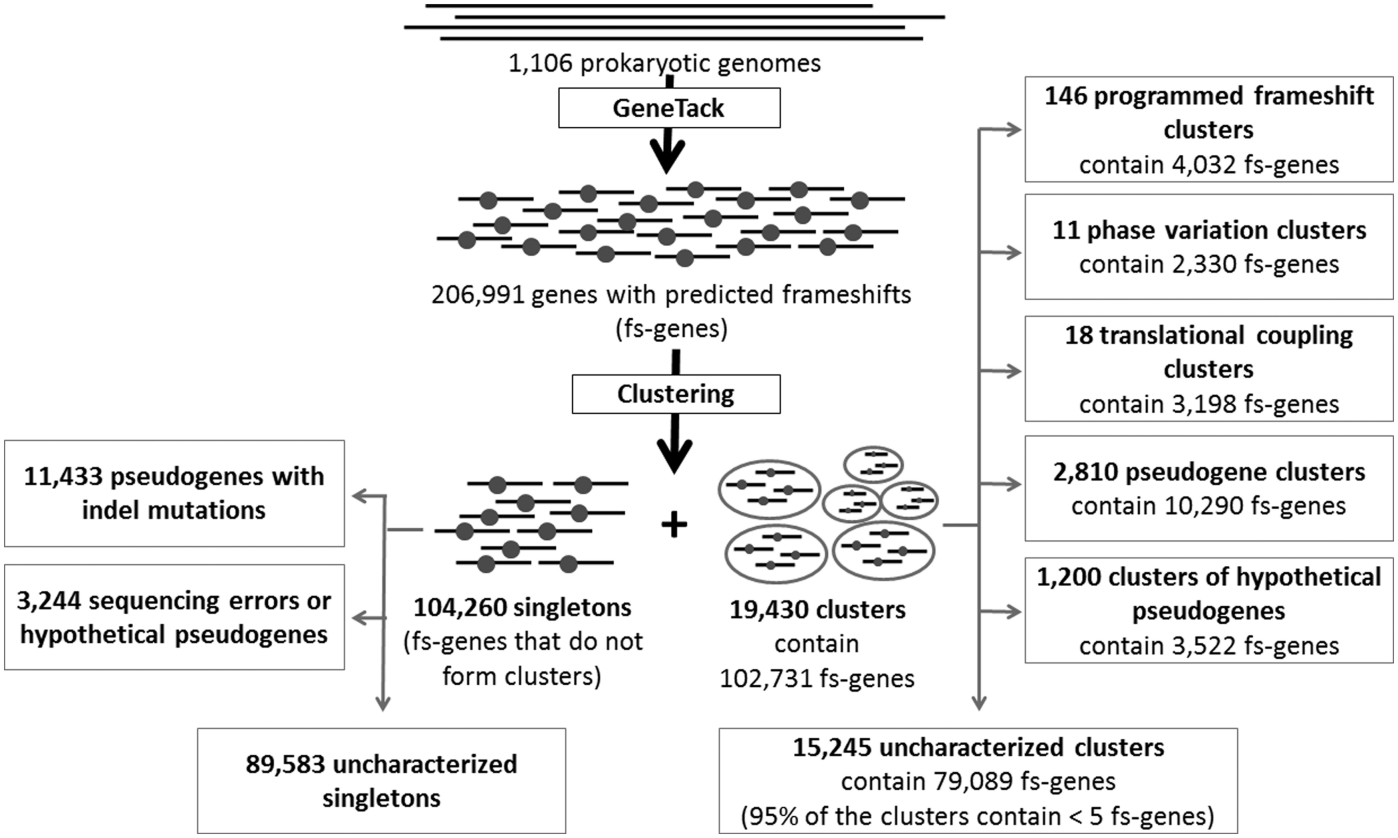
Classification of predicted frameshifts was done by using features specified in Table 1. One of the most important properties of a predicted fs-gene was its membership in a cluster. Singleton fs-genes (not orphan genes) are likely to be a result of indel mutation or sequencing error, while clustered fs-genes could represent programmed frameshifts, phase variation and translational coupling, as well as clusters of pseudogenes or genes with indel mutations.

We have found that 18 619 of the predicted fs-genes were annotated as pseudogenes and that 7186 of them belonged to clusters (3329 clusters with at least one *annotated* pseudogene). As annotation of pseudogenes may not be reliable, we excluded 411 clusters with fs-genes originated from three or more different genera (1361 fs-genes in total) assuming that real pseudogenes should be of relatively recent origin (72). Also, we excluded clusters of fs-genes with evolutionary conserved motifs in the frameshift boxes (potential clusters of fs-genes with *recoding*). We characterized the remaining 2810 clusters, each with at least one *annotated* pseudogene, as pseudogene clusters [10 290 fs-genes with 5484 fs-genes annotated as pseudogenes (Figure 5)]. Among the other 4806 fs-genes newly characterized as pseudogenes, many have appeared in genomes with no RefSeq annotated pseudogenes (Supplementary Figure S6).

Furthermore, considering fs-genes from some other clusters, we saw that predicted frameshifts should have truncated translation of a large part of an fs-gene (if not corrected by PRF or PTR). The fs-genes in these clusters did not exhibit features typical for *recoding* genes; they contained fs-genes from no more than two different genera, also, >50% of the cluster’s frameshifts were validated by BLASTp (Table 1). As there were no signs of PRF or PTR mechanisms present, disfunctional truncated protein products were likely to be produced. We characterized such clusters as clusters of hypothetical pseudogenes: 1200 clusters with 3522 fs-genes (Figure 5).

A ‘conserved pseudogene’ may sound as a misnomer; however, with promoter and transcription process intact, its transcript could carry regulatory functions at RNA level (73) thus keeping the ‘pseudogene’ under selective pressure. In fact, transcription and even translation of some pseudogenes have been demonstrated experimentally (74,75).

### Singletons

More than 50% of predicted fs-genes did not cluster; they formed a set of 104 260 singletons (Figure 5). Frame transitions in a singleton could be caused by sequencing error or by recent indel mutation; it may represent a false-positive fs-gene (pair of adjacent genes) as well as an orphan gene with *recoding*.

The set of singletons was divided into three groups: (i) those with frameshifts validated by BLASTp (∼30% of all singletons); (ii) those with two separate BLASTp hits to proteins in other species indicating a likely false positive (∼30%); (iii) those with no BLASTp hit to another protein in NCBI nr database, orphan fs-genes (∼40%). We expect that singletons of category (i) represent sequencing errors rather than genuine indel mutations. Singletons of category (iii) are expected to represent gene overlaps (33%), with the remaining 67% being divided between sequencing errors and indel mutations.

We saw above that 7186 of the whole set of 18 619 annotated pseudogenes were in clusters; on the other hand, the larger fraction of this set, 11 433 annotated pseudogenes (and predicted fs-genes) were singletons, indicating the rapid pace of pseudogenes degradation (76).

Frameshifts in 3244 singletons were confirmed by both BLASTp and Pfam; they were likely to be sequencing errors in functional genes or indel mutations in pseudogenes (see Figure 5).

Interestingly, some frameshift types are more frequent in specific locations of fs-genes (see Supplementary Materials: Distribution of relative frameshift coordinates). We observed elevation of frequency of frameshifts at the 3′-end of fs-genes (Supplementary Figure S7). One could speculate that indel mutations could truncate a gene slightly without affecting function of the protein product. Thus, a frameshift predicted close to 3′-end of a singleton would be more likely related to an indel mutation than to a sequencing error in comparison with other locations within fs-gene.

### Genomic distribution of programmed frameshift sites

Sequence motifs able to trigger frameshifts, ‘singular genomic elements’ (3), present at specific locations close to programmed frameshift sites should be avoided at other locations. Several authors analyzed frequencies of occurrences of frameshift-prone sequences within protein coding genes. In analysis of heptamer frequencies in *S. cerevisiae* genes, Shah et al. found known frameshift-prone sequences, C.TT_A.GT_T (77,78) and C.TT_A.GG_C (79), among the least frequent heptamers (80). Our analysis of the *E. coli* genome showed that the frameshift prone motif, A_AA.A_AA.G (65,66) is underrepresented (especially in highly expressed genes); however, it is not infrequent (57). A similar pattern was observed in *H. influenzae* and *Vibrio cholerae* genomes [the sets of highly expressed genes were taken from (81)]. Interestingly, poly-AT heptamers were present even in highly expressed genes, but the poly-AT heptamer frequency *ranking* computed for highly expressed genes was always lower than the poly-AT heptamer frequency *ranking* computed for a set of genes other than highly expressed genes (see Supplementary Table S5).

## DISCUSSION

Working on identification of fs-genes with programmed frameshifts we have grouped 4730 fs-genes into 146 clusters of candidate fs-genes with *recoding*. Using reporter genetic constructs based on the sequences of 20 *recoding* candidates, we confirmed that the clusters were enriched with *recoding* genes by exploring frameshifting *in vivo*. We have identified four new families of fs-genes with programmed frameshifts: fs-genes for Magnesium chelatase, Spore germination protein, DUF111 and DUF772.

While the approach to cluster characterization using multiple features (Table 1) produced a number of interesting results, the nature of frame transitions in many large clusters remained uncharacterized.

Conservation of co-location of overlapping/adjacent coding regions indicates functional relationship (82); still, the likely co-regulation of these gene pairs may use different mechanisms even in homologous genes from the same cluster. Indeed, in some experiments we observed evidence of frameshifting along with evidence of initiation at the downstream coding region, suggesting that translational coupling and *recoding* mechanisms are not mutually exclusive but rather interchangeable. Thus, the task of unambiguous characterization of the nature of frame transition for a whole cluster may not be correctly stated.

### Why does most programmed frameshifting occur in mobile elements?

A number of the largest programmed frameshift clusters were clusters of transposase fs-genes. The fact that the programmed frameshifting is so frequently used to regulate the gene expression in mobile elements is an intriguing but not entirely new observation. Contributing to selective advantage of programmed frameshifting is the fact that it is an economical gene expression regulation mechanism encoded inside the mobile element. A low, 1–3%, frameshifting efficiency moderated by stimulators around the frameshift sites results in low level of protein product. This may provide selective advantage for transposases because active fs-gene expression would result in frequent translocation of mobile elements, potentially harmful for their hosts. The low expression level would allow maintenance of a balance between proliferating/translocating and host survival (83). The poly-A slippery sequence characteristic for programmed frameshifting in transposases genes can be used in both PTR and PRF (14). Our data on transposase families where the frameshift direction (+1 or −1) is not conserved suggest that PTR is more likely to occur in this case (e.g. transposase families HTH_Tnp_1, DDE_Tnp_1, HTH_Tnp_IS630).

Interestingly, it has been shown recently that specific translation pausing during ribosomal frameshifting contributes to the preference of a transposase for acting on the IS element from which it is expressed (84). This subtle mechanism could be an additional selection force that maintains utilization of *recoding*, as the *cis*-acting transposase promotes propagation of its own mobile element and not another IS element responsive to the same transposase.

### Many genes with frameshifts have incorrect annotations

Genes with frameshifts present a difficulty for standard annotation procedures. Many are incorrectly annotated either structurally or functionally. Particularly, genes with indel frameshift mutations might be annotated as two separate adjacent genes (often both genes are annotated as hypothetical genes). It is difficult to discriminate between frameshifts due to indel mutations and frameshifts related to *recoding*. Notably, several well-known genes with *recoding* were either not annotated in some genomes or annotated as pseudogenes, e.g. 17 out of 428 *prfB* genes. For these 17 *prfB* genes, a manual inspection has shown that all of them had intact programmed frameshift signals and did not have any other frameshifts or premature stop codons to justify pseudogene annotation. In the protein database, some protein sequences of Release Factor 2 were missing N-terminal ends because of wrong annotation of *prfB* genes (e.g. *Lactobacillus johnsonii NCC 533*, NC_005362). In total, out of 4302 fs-genes with predicted programmed frameshifts, 611 were annotated as pseudogenes. We are certain that at least some of them, like the *prfB* genes, were erroneously annotated. In general, due to the lack of universal methods for identification of *recoding* instances and classification of frameshifted genes, it is likely that erroneous annotations will continue to appear in databases. Nonetheless, we hope that the resource developed in the course of this research work, particularly the clusters of the fs-genes and the web-based tools of fs-gene prediction and classification will help improve annotation of frameshifted genes.

### Uncharacterized clusters

In this work, we provide characterization of 38 319 fs-genes (23 642 clustered fs-genes and 14 677 singletons); this number constitutes only 19% of all predictions. Notably, 79 089 fs-genes belong to still uncharacterized clusters (Figure 5). The existence of homologous fs-genes makes it more likely that the frame transition, predicted as a frameshift, has a non-trivial biological meaning even if the transition happened between a pair of genes, not inside a single gene. These gene pairs might participate in a biological process (56) encoding functionally related proteins. Some of these gene pairs could be regulated by translational coupling, some could be formed by gene fission, etc.

The task of characterization may lead to discovery of new patterns of regulation, metabolic pathways and protein complexes. Notably, we may have missed some clusters of fs-genes with *recoding* because the 146 clusters we focused on were selected based on a limited set of the most prominent programmed frameshift motifs.

All the fs-genes predicted in this study were included in the GeneTack database (85). As of February 2013, there were 2294 genomic sequences longer than 1 Mb in the RefSeq database; 1188 new genomes have been added since the start of this project. New genomic data to be included into expanded clusters should help identify functional roles of yet uncharacterized fs-genes as well as new evolutionary conserved frame transitions.

## AVAILABILITY

Additional information about fs-genes and clusters is available in the GeneTack database at http://topaz.gatech.edu/GeneTack.

## SUPPLEMENTARY DATA

Supplementary Data are available at NAR Online: Supplementary Tables 1–5 and Supplementary Figures 1–7.

## FUNDING

The work of I.A. and M.B. was supported in part by the USA National Institute of Health grant [HG000783 to M.B.]; the work of P.V.B., A.C. and J.F.A. was supported in part by the Wellcome Trust grant [094423 to P.V.B.] and by Science Foundation Ireland grant [08/IN.1/B1889 to J.F.A.]. Funding for open access charge: The Wellcome Trust grant [094423 to P.V.B.].

*Conﬂict of interest statement*. None declared.

## Supplementary Material

Supplementary Data
