# Supplementary material for: Identification of the nature of reading frame transitions observed in prokaryotic genomes
Source: Nucleic Acids Res. 2013 May 6;41(13):6514–30. doi: 10.1093/nar/gkt274 (PMC3711429; doi:10.1093/nar/gkt274)
Supplement: Supplementary Data [file supp_41_13_6514__index.html]

Identification of the nature of reading frame transitions observed in prokaryotic genomes — Supplementary Data 

# Identification of the nature of reading frame transitions observed in prokaryotic genomes

## Supplementary Data

files

**Files in this Data Supplement:**

- Supplementary Data - pdf file
