# Supplementary material for: Identification of the nature of reading frame transitions observed in prokaryotic genomes
Source: Nucleic Acids Res. 2013 May 6;41(13):6514–30. doi: 10.1093/nar/gkt274 (PMC3711429; doi:10.1093/nar/gkt274)
Supplement: Supplementary Data [file supp_gkt274_nar-02973-n-2012-File002.pdf]

Supplementary Figures/Tables

Identification of the Nature of Reading Frame Transitions Observed in Prokaryotic Genomes

Ivan Antonov, Arthur Coakley, John F. Atkins, Pavel V. Baranov and Mark Borodovsky

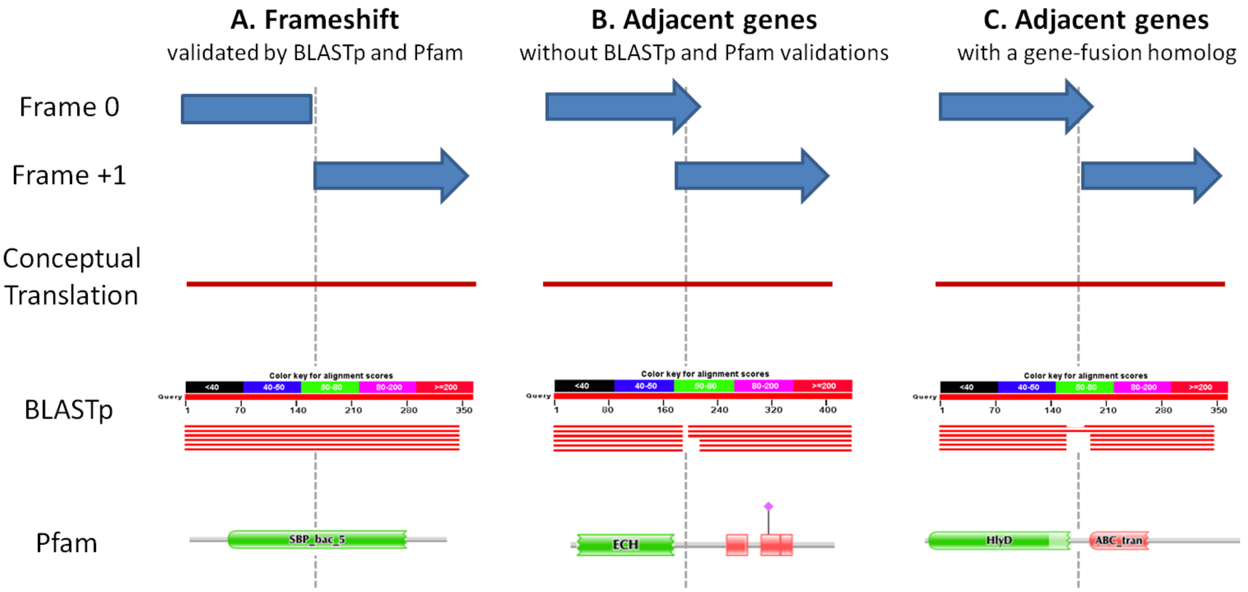

Figure S1 Possible outcomes of the BLASTp and Pfam searches for a conceptual translation of fs-gene used as a query. If a frameshift position is covered by BLASTp hit (or Pfam domain) the predicted frameshift is considered to be validated by BLASTp (or Pfam).

Figure S2 Number of frameshifts predicted in a prokaryotic genome correlates with genome length (data from analysis of 1,106 genomes). Total number of predicted frameshifts was 206,991. Note that genomes shorter than 1Mb were not considered.

A.

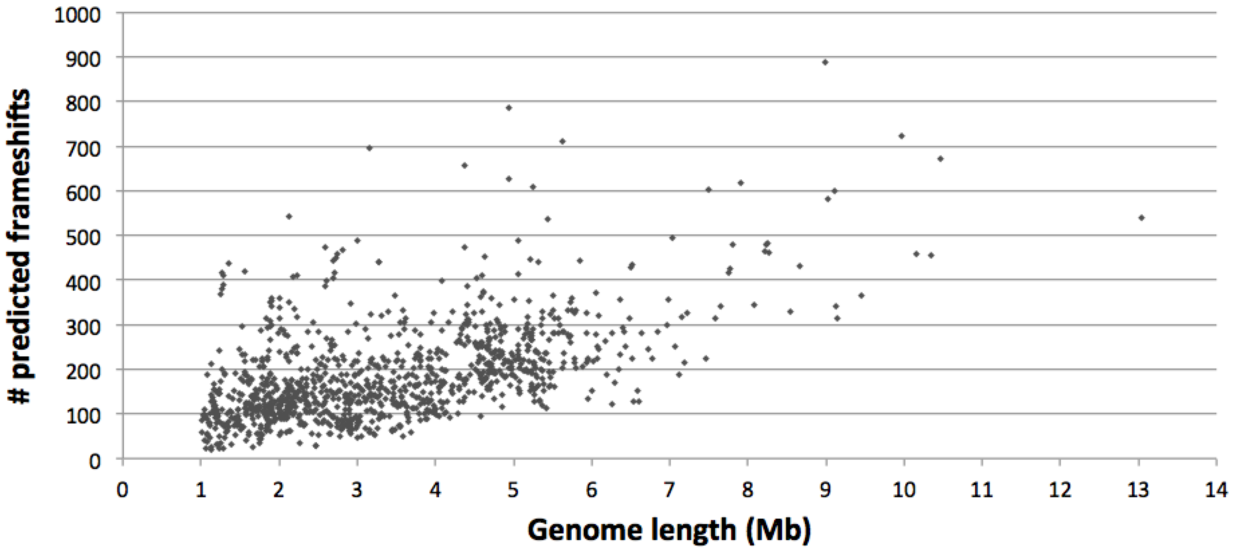

B. Genomes that significantly deviate from the linear regression line line:

Genome	Length (Mb)	GC%	Observed FS	Predicted from regression	Diff
Sodalis glossinidius	4.2	55%	1707	215	1492
Xanthomonas oryzae	4.9	64%	785	242	543
Renibacterium salmoninarum	3.2	56%	695	179	516
Frankia sp.	9.0	71%	889	384	505
Mycobacterium ulcerans	5.6	65%	710	266	444
Orientia tsutsugamushi	2.1	31%	543	143	400
Xanthomonas oryzae pv.	4.9	64%	627	242	385
Xanthomonas oryzae	5.2	64%	610	252	358
Rickettsia massiliae	1.4	33%	438	116	322

Figure S3 Alignment of frameshift vicinity sequence of *Thermus thermophilus* (for which transcriptional realignment was shown experimentally) with poly-A motifs from 9 fs-genes of DNA polymerase III cluster.

```
Thermus_thermophilus*               -------GAGG----GAGAAAAAAAAAGCCTGA------- 22
Chlorobium_luteolum                 ----CAGGAGCCT-CAAAAAAAAAAGCCCCTGATG----- 30
Chlorobium_tepidum                  ---TCGGCGGACG--AAAAAAAAAAGCT--TGAGCCT--- 30
Flavobacterium_johnsoniae           ACTTTGATAGA----AAAAAAAAAGTTGAGCAAT------ 30
Flavobacterium_psychrophilum        CTTTTGATAGA----AAAAAAAAAGCTAAACAAT------ 30
Coraliomargarita_akajimensis        ---GGTGCGGACG-AAAAAAAAAAGTCCAGTGAT------ 30
Cytophaga_hutchinsonii              ----TGGAAGACC-TAAAAAAAAAAGTAAATAAAC----- 30
Capnocytophaga_ochracea             ----TGAGGGGGA-TAAAAAAAAAA-----TGATGGACTT 30
Prosthecochloris_aestuarii          -----GGCCGCCGGTAAAAAAAAAAGCCCCTGAAC----- 30
Pelodictyon_phaeoclathratiform      ----CTGCAGGCT-CAAAAAAAAAAGCACCTGACA----- 30
                                             *      * ******        *
```

Figure S4 Western blot analysis and quantification of frameshift products. Bacteria strain MG1655ΔlacIZ harboring reporter plasmids encoding for GST (in absence of frameshift) and/or GST-MBP (in presence of frameshift) were diluted 1:100 in LB broth, cultivated during 2 hours, induced with 100mM IPTG for an additional 2 hours. Immunoblots were incubated at 4°C overnight in 5% milk/PBS-Tween containing a 1:500 dilution of rabbit anti-GST. Immunoreactive bands were detected on membranes after incubation with appropriate fluorescently labeled secondary antibodies. Bands corresponding to the termination product and frameshift product are shown. Sequence information can be found in GeneTack database by a search for Frameshift ID.

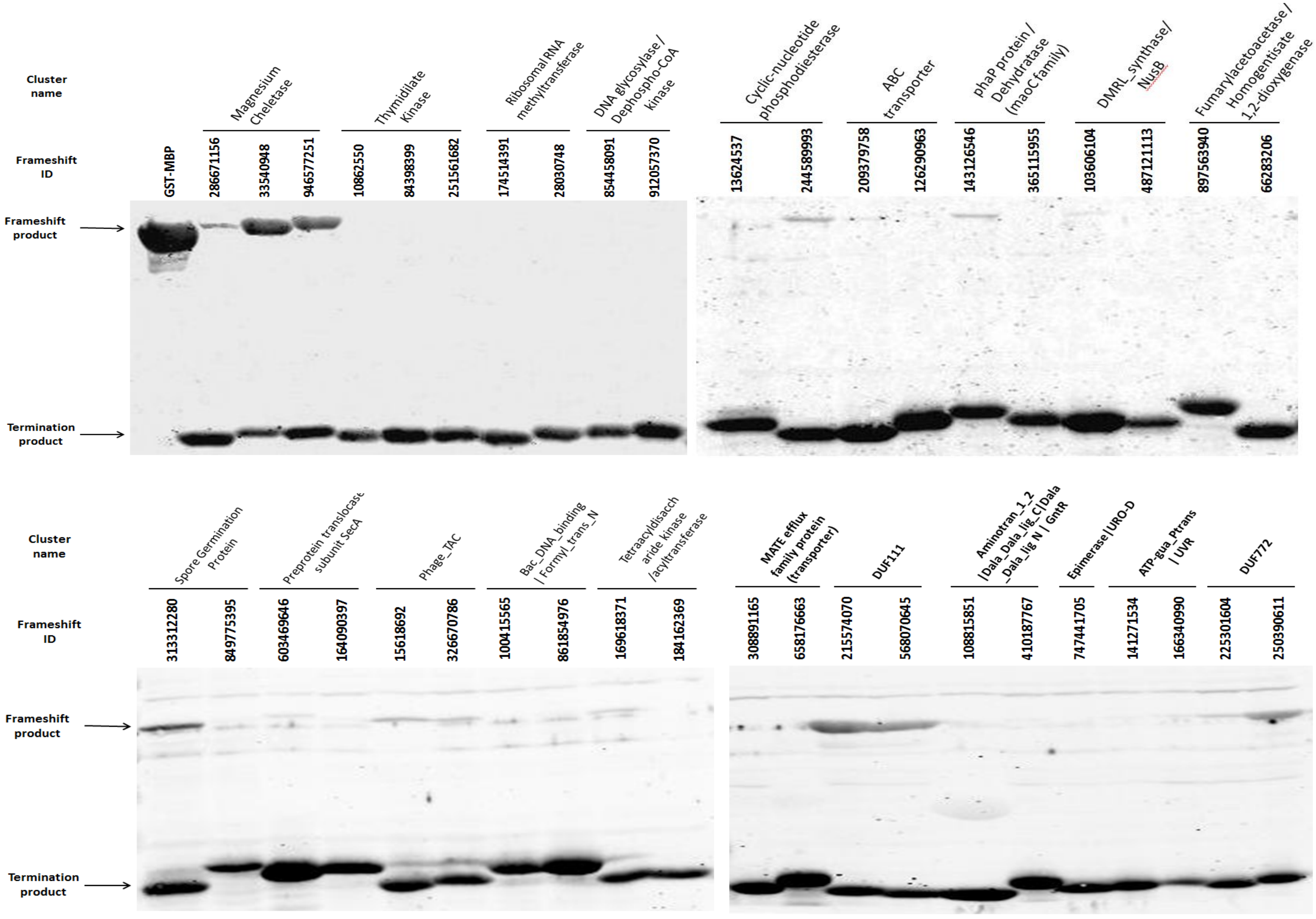

Figure S5 Anti-his western blot analysis of frameshift products and internal initiation (translational coupling) products. Bacteria strain MG1655ΔlacIZ harbouring reporter plasmids encoding for GST (in absence of frameshift) and/or GST-MBP (in presence of frameshift) were diluted 1:100 in LB broth, cultivated during 2 hours, induced with 100mM IPTG for an additional 2 hours. Immunoblots were incubated at 4°C overnight in 5% milk/PBS-Tween containing a 1:2000 dilution of rabbit anti-HIS. Immunoreactive bands were detected on membranes after incubation with appropriate fluorescently labeled secondary antibodies. Bands corresponding to the termination product and to internal initiation (translational coupling) products are shown.

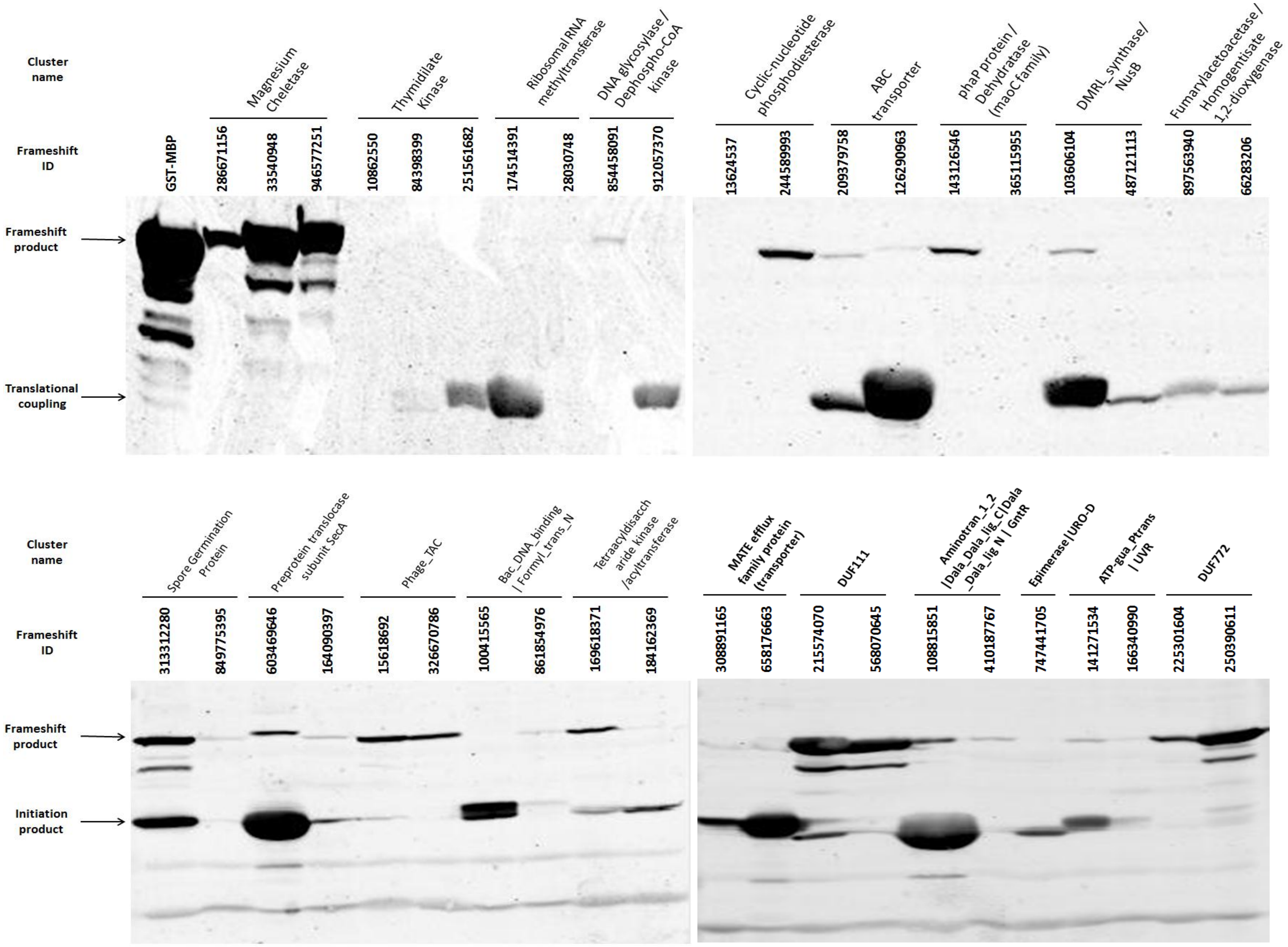

Figure S6 Distribution of the number of annotated and predicted pseudogenes among prokaryotic genomes. Black bars reflect pseudogenes annotated in RefSeq. The white bars show the updated distribution with 4,806 pseudogenes identified in this work and added to the annotated pseudogenes. The largest change in distribution has been observed for genomes with less than 10 pseudogenes annotated in RefSeq. Hypothetical pseudogenes are not included into the set of "New pseudogenes".

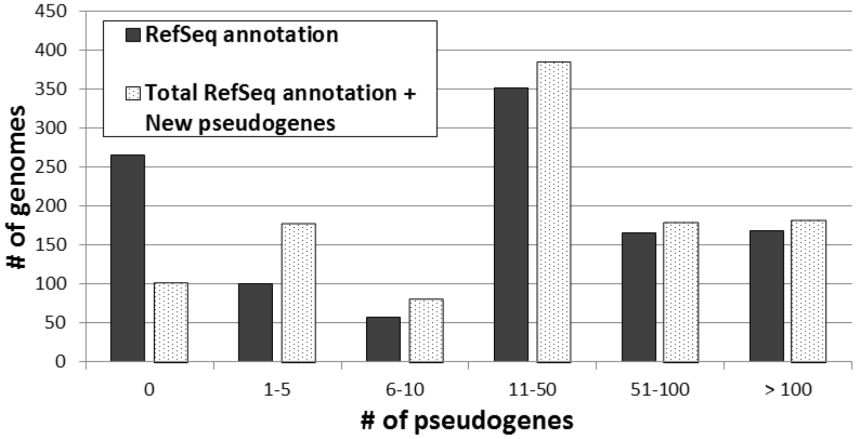

Distribution of relative frameshift coordinates in fs-genes

The frameshift coordinates normalized to the length of a predicted fs-gene had a distribution with characteristic shape (Figure S7). The distribution represents the whole set of frameshifts including i) those caused by sequencing errors and indel mutations; ii) frameshifts predicted at overlaps of adjacent genes; iii) programmed frameshifts. We assume that these three groups produce distributions with distinct shapes. Coordinates of frameshifts related to sequencing errors and indel mutations are assumed to follow a uniform distribution. The coordinate of GeneTack prediction at a gene overlap was assumed to be modeled by the function

$$r = \frac{x+t}{x+y-z},$$

where *x, y* are lengths of adjacent genes X and Y, *t* is the error in detecting the frameshift position and *z* is the length of gene overlap (positive if X and Y overlap and negative otherwise). The distribution of value *t* was determined from GeneTack predictions for artificial fs-genes in *E. coli* (Figure 7C); distribution of *z* value was derived from all the predicted fs-genes. The X and Y gene lengths, x and y respectively, were assumed to follow a gamma distribution with the scale parameter equal to 100 codons. Distributions of x, y, t, and z were used for computational modeling of distribution f(r).

The observed empirical distribution of the relative frameshift coordinate θ could be represented as the sum of uniform distribution *U* with density 1, the *f(r)* distribution and the distribution *G* of the frameshift coordinates related to the programmed frameshifts (Figure S7B).

Elevation of frequency of predicted frameshifts at the end of (0,1) interval, at fs-gene 3' ends, could be related to mutations which, even though they lead to premature stop codons, do not affect protein function and remain in the population. This is in contrast to mutations that occur in the middle, or in the beginning, of a gene. The distribution drops to zero early at both ends can be considered as artifacts, since frameshifts predicted within 50nt distance from fs-gene borders were discarded. The horizontal dashed line provides a rough division between flat (uniform) probability mass function related to random frameshifts, i.e. sequencing errors and indel mutations, and the bell shaped distribution related to frameshifts predicted at gene overlaps; this bell shaped distribution is "decorated" with spikes related to programmed frameshifts in large clusters of *recoding* genes as well as other clusters where evolutionary conservation of frameshift positions was observed.

Figure S7 (**A**) Empirical distributions of frameshift coordinates relative to fs-gene lengths for i/) all the predicted frameshifts (206,991 fs-genes), ii/) singletons (104,260 fs-genes) and iii/) frameshifts in clusters containing 10 or more members (47,278 fs-genes in total). (**B**) Distribution of relative coordinates of all the predicted frameshifts i/ is shown along with the theoretical distribution combining a uniform distribution of coordinates of non-clustered frameshifts and distribution of co-ordinates of false positive predictions (the (x+t)/(x+y-z) distribution – see text). The non-clustered frameshifts correspond to indel mutations and sequencing errors while the false positives are predicted mostly at junctions of adjacent genes (with overlapping ORFs). The theoretical curve fits best to the empirical distribution if α= 0.005. See text for more details.

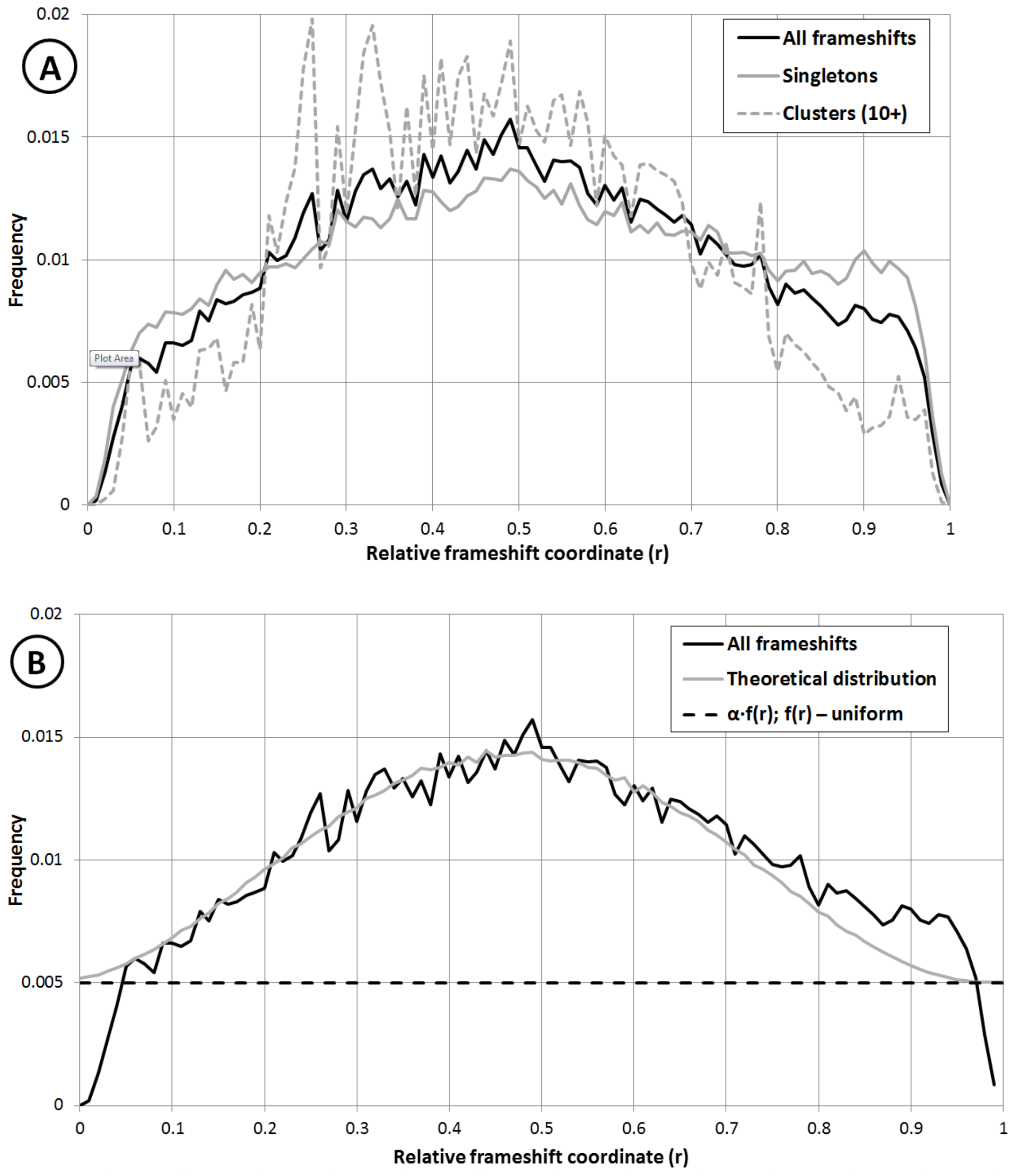

Figure S7 (C) Distribution of frequencies of offsets in predicting frameshift positions. In order to get data for this distribution we have applied GeneTack to 400 *E. coli* genes (longer than 1000 nt) with a single frameshift created at random in a position separated by at least 150nt from the gene border. The program successfully predicted 351 frameshifts (the remaining 49 frameshifts were predicted as separate genes). The distribution shows that in 83% of cases a prediction is located within 5 nt from the true frameshift position.

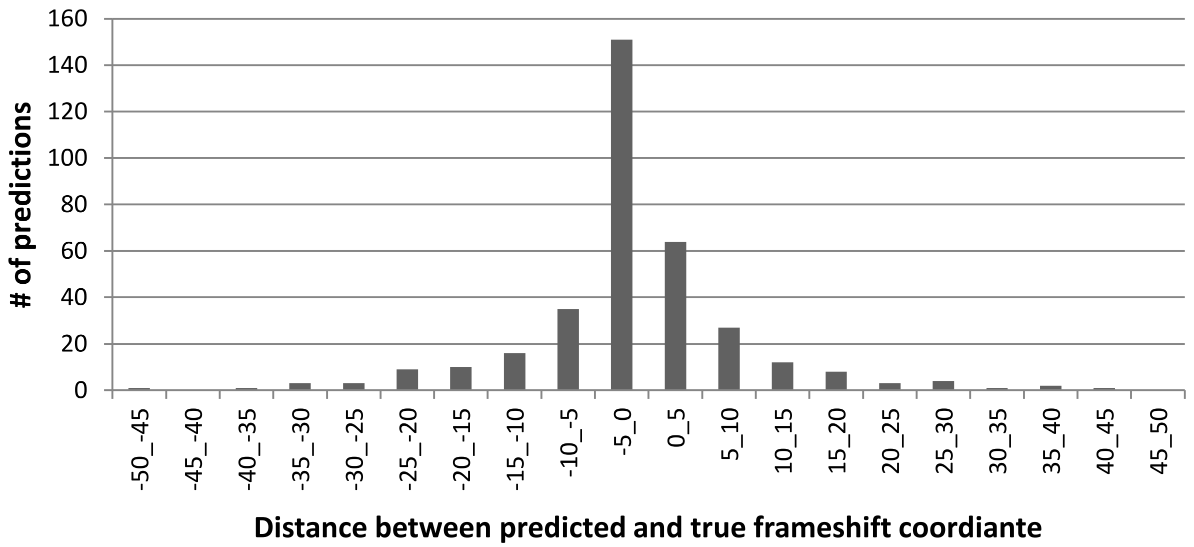

Table S1 The lists of high scoring heptamers used to select the 7 A-rich motifs (shown in bold) that could cause programmed frameshifting. Also shown in bold is the *prfB* motif CTT_TGA_C.

Rank	+1 heptamer	Score
1	GTG_CGC_G	594.3
**2**	**CTT_TGA_C**	**428**
3	GAC_GAG_G	326.04
4	CTG_GAA_A	299.57
5	TGG_CGC_G	280.16
6	**AAA_AAA_A**	**247.87**
7	AAA_GAG_G	195.07
8	**AAA_AAA_T**	**193.47**
9	CTT_TCC_A	175.86
10	GTC_ATC_G	129.79

Rank	-1 heptamer	Score
1	**A_AAA_AAG**	**2105.72**
2	**T_AAA_AAA**	**496.83**
3	T_CAA_TTA	289
4	**A_AAA_AAC**	**231.05**
5	C_CTG_CCG	198.45
6	**A_AAA_AAA**	**79.84**
7	G_CGC_GGC	72.87
8	G_GAG_GCA	69.74
…	…	…
30	**G_AAA_AAA**	**26.53**

Table S2 Inserts cloned in between GST and MBP genes. The sequences were derived from the frameshift vicinity of the corresponding fs-genes. The actual insert sequence also included flanking 18nt upstream and 45nt downstream sequences. Underscores indicate frame of the ORF1. FS % -- frameshifting efficiency detected in experiments.

Cluster name [Cluster_ID]	Frameshift ID	INSERT SEQUENCE	FS %
Magnesium chelatase [131733585]	33540948	AAA_AAG_GAA_AAT_AAT_GAC_GT[A_AAA_AAA_AAA_A]CA_AGA_ATA_AAT_TAA_ATA_ATG_AAT_CAA_ATA_ATA_ATG_AGA_ATA_ATA_AT	63% ±4.36
	286671156	AAG_GTG_GGA_GCG_CCC_GCC_AC[A_AAA_AAA]_GCC_TGA_GCC_CCC_GCG_GCC_CCG_CGC_CAC_AGG_CAA_GGG_CTG_CGG_GGG_	10% ±6.25
	946577251	GAG_CAA_AAA_AAT_GAT_GAC_GT[A_AAA_AAA_AAC_A]TG_ATG_AAA_TAA_GAA_ATG_AGT_TTG_AAG_AAG_AAA_ATG_AGG_ATT_CAA_AT	40% ±1.73
Thymidylate kinase [984773919]	10862550	GAT_TAT_CTG_AAA_GTG_CTT_[AAG_GAA_AAA_AAT]_GCG_CAG_TAA_GTA_TAT_CGT_CAT_TGA_GGG_GCT_GGA_AGG_CGC_AGG_CAA	
	84398399	T_ACG_CGG_GCA_TCC_GAA_CCG_[AAA_AAA_AAG]_CAA_TGA_ACC_CAG_CAA_TGA_CCG_AGC_ATT_CAT_GAA_CGC_ACC_CGG_AAT	
	251561682	AAG_GTA_TAC_CGC_TCA_GCG_T[TA_AAG_GAA_CGG_GAT_GAA_CAG_TAA]_ATT_TAT_CGT_CAT_TGA_AGG_ATT_GGA_AGG_CGC_AGG _GAA_GAC_CAC_GGC_	
Ribosomal RNA methyltransferase [884136395]	28030748	G_ACG_CTG_CCC_GGT_TCG_CAC_[GAG_GGC_TTC_GAC_GGC_GGC_TTC_GCC_GCA_CGG_TTC_CGC_AAA_ACG_GAG_GGG_TAA]_AGA_ TGC_CAG_AAC_GCG_AGG_AGC_CGT_GGC_GGA_AAA_CGC_TCG_AAA_CCT_	
	174514391	GGA_TTC_TTT_ATT_AGC_AAG_AT[A_AAA_AGA_AAG_GAA_AG]T_TAG_AGA_TGA_TAA_CTG_CAG_AAA_AAA_AGA_AGA_GAA_ACA_AAT_TCT_TAC	
DNA glycosylase / Dephospho-CoA kinase [522343807]	854458091	T_ATT_GTC_AAA_TTA_AAA_GTT_[GGA_GGA_AGG_GGG_ACG_CAC_ATT_TGT_CCA_AAA_TGC_CAG_AAA_AAA_AGA]_CCA_TGA_TTA_ TTG_GCA_TCA_CAG_GAG_GCA_TTG_CAT_CAG_GAA_AAT_CGA_	Low
	912057370	CCA_CGT_TGT_GCA_ACG_CCG_AT[T_GAA_AAA_ATC_AAA_GTA_GGA_GGT_CGT_GGG_ACA_CAC_CTT_TGT_CCT_GCA_TGT_CAA_AAA_ CGA_TGA]_CAA_GAA_TTA_TTG_GAA_TCA_CGG_GCG_GAA_TTG_CTT_CGG_GAA_AAT_CAA_	
Cyclic-nucleotide phosphodiesterase [430699271]	13624537	TAC_CAG_TTA_ATC_CCG_ATC_AA[T_TTA_AAG_AAA_AAA]_TTG_AGA_AAG_CTG_ATG_GCA_CTC_GCG_AGC_ACG_TCT_TTT_ATA_CCC_AAG	
	244589993	GCT_CAT_ATG_CAT_AAG_CTT_GT[A_AAA_AAG]_AAG_TTG_TAA_ATG_GTG_TCA_TTA_TCA_CGG_AAC_CAG_ATA_AAT_ATG_GAA	6% ±2.08
ABC transporter [309851863]	126290963	AAA_ATA_CGA_CAA_AAA_ATC_T[TA_ATT_TCA_AAA_CGA_GGT_GAG_GAA_AAT_GGG_ACT_TAC_AGC_AAC_TGA]_AGC_ACT_CAT_GAA_ AAT_GAA_GGA_AAA_AGG_CTA_TAA_ACA_CAC_AGA_TAA_	Low
	209379758	A_ATT_GGT_GCG_ATT_TTA_TGG_[AAG_AAG_AAA_AAA_A]CT_GCA_TAA_AGT_AGG_GAT_AGA_TAT_GAA_TCT_AAC_AGA_AGC_TTT_ACG_CC	5% ±3.61
phaP protein / Dehydratase (maoC family) [720147899]	143126546	CTT_CAA_AAA_CAA_TTA_GAT_GA[T_TTT_TTG_ACG_GAG_TTC_AAG_TCT_ACA_CAA_CTG_GAA_CTT_GTA_AAA_AAG]_TTC_GAG_GAA_ AAC_TCC_AAA_AAT_CTA_TTT_ACT_TCC_ATC_AAA_TAA_GAA_	6% ±1.16
	365115955	AAG_TCC_AAA_CAA_CTA_GAA_CT[C_GCA_AAG_CAG_TTC_GAG_GAA_AAC_TCA_AAA_AAT]_CTA_TTT_ACT_TCC_ATC_AAG_TAA_GAA_ AAA_TGT_GGC_AAC_TAA_CTG_CAG_	
DMRL_synthase | NusB [310905921]	103606104	GAC_AAG_GAC_AAG_GGT_GGC_TT[T_GCA_GCC_CGT_GCC_GCA_TTG_ACT_ATG_ATC_GGC_CTG_CGC_AAA_AAA_TTC_GGA_GCC_TGA_ TCG_TTA_TGA]_ATT_CTA_TCC_CTG_AAG_GCC_GTC_CGA_CTC_CCA_ATC_TGC_CGC_GAA_CTG_	1% ±0.23

	487121113	CAG_ACC_AAG_GGA_GTT_GGG_TT[T_AAA_CCT_GCC_TCC_GAC_TCG_TAC_CGG_CTC_GCC_TGA]_TTT_CAA_CAA_CGG_ATC_CCT_CGC_<br>TCA_TGA_CCT_CCA_AAG_ACG_CCC_CGA_	
Fumarylacetoacetase / Homogentisate 1,2-dioxygenase [645374543]	66283206	G_CAT_GTT_TTA_TCG_TCA_CAT_[GGG_GGA_G]CT_ACC_TCA_TAA_ACG_ACA_TGT_ACA_ATT_CCG_TAA_AAA_AGA_CGG_GTC_GC	
	897563940	T_ACA_TTA_CGT_AAT_ACG_GTC_[AAA_AAA_GAA_AAG_GAA_GCA_GGT_GAT_GAG_CAT_GTT_TTA_TCG_TCA_CAT_GGG_GGA_GCT_ACC_TCA_TAA]_<br>ACG_ACA_TGT_ACA_ATT_CCG_TAA_AAA_AGA_TGG_ATC_GCT_TTA_TCG_TGA_	

Cluster Name	Frameshift ID	Insert Sequence	FS %
Spore Germination Protein [447662180]	313312280	CTA_TTT_ATT_ATT_GTG_TGG_GTG_AAA_AAA_AAA_TGG_AAA_AAA_CCA_AAA_GAA_AAT_TAA_TAC_TTC_TTA_TTT_GCA_TCA_GTG_TCT_TCA_GTC_TAA_CTG_GTT_GTT	13% ±3.09
	849775395	CGC_TTG_TTC_GGA_TTC_AAT_CTA_CGT_CAT_TCG_TTG_TAT_TGG_ATT_ATA_CCC_GTG_ATC_TAT_GTA_GCC_TCC_TTG_TCG_CTG_CTT_TCC_AGA_CAG_CAG_ATG_AGT_CGG_ATG_ACG_ACT_ATT_TAC_TCG_CAT_ATA_ACT_CTG_TAC_ATT_ATT_TAC_GTG_TAC_CCC_TAT_TTT_TTG_TAC	4% ±1.56
Preprotein translocase subunit SecA [970108792]	603469646	AGA_AAA_AAA_GCC_CCT_GTA_GTA_AAG_GAA_AAA_GAG_GCT_GGG_AGA_AAC_GAC_CCA_TGT_CCA_TGT_GGT_AGT_GGG_AAG_AAA_TAC_AAA_AAA_TGT_TGT_GGA_GAG_TAA_GGC_ATC_TTC_AAA_ATT_AAG_AGG_TGA_ATG_TAT_GTT_AAA_TTT_AGA_TAT	3% ±0.60
	164090397	AAG_CGT_CAA_CCT_GTG_CGT_GTA_GAT_AAA_AAA_GTG_GGC_CGT_AAT_GAT_TTA_TGC_CCA_TGC_GGA_AGT_GGC_AAA_AAA_TTC_AAA_AAT_TGT_CAT_GGA_AGA_AAC_GCT_TAA_TTA	-
Phage_TAC [862991913]	15618692	GCT_GAT_GCA_GAG_TCG_GCC_AGA_AAA_AAG_TAG_CCC_GCC_CGG_AAA_TTC_GCT_TTC_TGA_TGC_GAC_TTG_CGC_TCC_GTC_TGG	7% ±0.79
	326670786	GGA_ATG_AGT_CAG_GAA_GAA_GCG_GGA_AAG_CCG_TAA_AGC_AGC_CGC_TGA_CCT_TCT_TTC_TGC_TGT_CAC_TGG_CGC_TCC_GGC_TGG	6% ±0.61
Bac_DNA_binding | Formyl_trans_N [931215581]	100415565	CGA_AAC_AAT_AAG_GCA_GTT_TTA_GAT_GAG_CAA_GAA_CTT_CCA_GAA_TCT_GGT_TAT_GCA_AAC_GAC_TAA_GCC_TTT_CGC_GGT_AGG_CGC_ACT_GTT_AGC_GAT_TTC_GCT_TTC_ATG_TGA	6% ±4.09
	861854976	ACT_ATG_GAA_GCA_GAT_TAT_GCA_GTT_CTT_GAC_GAA_ACA_AAA_CTT_CCT_GCA_CAC_GGC_GCG_CAG_TAC_CCA_CAA_TAA_AAA_AAT_AAG_TGC_CCT_GCT_ATT_ATG_TGT_GGG_CAC_TTT_ACT_TCC_AAC	3% ±1.44
Tetraacyldisaccharide kinase /acyltransferase [181800409]	169618371	ATA_AAC_CAT_CCT_GAT_TTA_TTA_AAT_GAA_AAA_ATT_TTT_AAA_AAA_GCT_TAG_ATA_TTT_AAT_TGA_GTA_TTT_TAT_CGT_TGT_TAT_ATT_TCT_GAA_AGT_AAT	7% ±2.45
	184162369	ACA_AAC_AAA_CTA_ATT_AAA_TTA_AAT_GAA_AAA_AAT_TAA_ATA_TTT_TTT_TGA_ATT_TTT_AAT_TAT_ATC_TTC_TCT_TTT_TAT_TAT_TTA	-
MATE efflux family protein (transporter) [523977875]	308891165	AAT_TCA_TAT_AGA_TGG_AAA_AGT_AAA_AAA_TGG_CAA_GGC_AAA_GCA_CTG_GTA_TAA_AAG_GAA_TTT_TAG_AAT_TTA_AAG_AAT_TTC_GTT_TTG_CTT_TTG_AAA_AGA	-
	658176663	TGG_ATA_GGA_TTT_TGT_GCA_GAT_GAG_TTT_TTG_CGT_GGA_ATG_GTA_AAT_TCA_TAC_AGA_TGG_AAA_AGC_AAA_AAA_TGG_CAA_GGC_AAA_GCT_CTA_GTT_TAA_AAT_CAT_TTA_TTT_ATA_AAG_ATG_AGT_GCT_ATT_TTT_ATT_CTA_AAA_AAC	-
DUF111 [782478235]	215574070	ATA_CTC_AGA_ACT_GTA_TTA_GGT_GAA_AAA_AAA_AAG_AAG_AAT_TAA_TTT_ATG_AAA_TTA_GTG_CAA_ATA_TTG_ATG_ATA_TGT_CTT_CAG_AAA_TCT	39% ±5.32
	568070645	AAT_ATA_GTA_AGA_GCA_ATC_ATA_GGA_AAA_AAA_AAC_TAA_ACT_TGA_GTA_GCA_AAT_ACT_TTG_AGA_TAT_TTG_CTA_ACG_TTG_ATG_ATA	34% ±5.91
Aminotran_1_2 |Dala_Dala_lig_C|Dala_Dala_lig N | GntR [392008946]	108815851	AAA_ATA_ATT_GGT_GAT_ACA_TTA_AAA_AAT_GAA_ATT_TGG_AGT_TGA_GAA_AAT_GAA_AAT_CGG_TGT_TAT_TAT_GGG_CGG_GGT_ATC_GTC_TGA_AAA	-
	410187767	CGC_AGC_GAC_CTG_GAG_CGG_GGC_GCC_CGG_CGG_GGT_GCC_CGG_CGC_GGC_ACC_CCG_GCC_GGA_ACG_GAC_CGC_TAG_GGG_GGC_CCT_CGT_GGT_GCG_GCT_GGC_GGT_CCT_GCG_CGG_CGG_GCA_CTC	6% ±5.21

Epimerase|URO-D [655521599]	747441705	GAA_CAC_CTG_AAA_GCA_TTG_GTT_GAC_TTC_ATC_AAA_GAA_GAA_AGC_GGC_AAA_TAT_CAT_CAA_GCT_TAA_AAA_TTT_TTA_AAA_TTA_CCG_GCG_AAT_CCA_TGA_ATA_TTA_TCG_AAA_CCC	-
ATP-gua_Ptrans | UVR [786465964]	141271534	CGT_GAT_CAG_ATT_AAT_CAG_CTA_AAA_AAT_CAG_AAT_ACT_ACC_GAT_GCT_CCC_TAA_TCA_TAT_TCT_TAC_TGC_TAT_CGC_AAC_GAT_CAA_GCA_TTC_TTT_GAG_AAC	8% ±5.41
	166340990	CGA_GAC_CAG_ATT_AAT_CAT_TTA_AAA_AAT_CAG_AAT_TCG_CAT_GAT_TCT_TCC_CAA_TGA_CTT_ACT_TCT_TAA_TTT_TGC_TAG_TAA_GAA_AGA_CGC_CCC_TCC_TAC_AAA	-
DUF772 [621432021]	225301604	GCT_GCG_GTA_ATC_GAA_GAT_CGT_GAG_GTA_CAT_GGA_AAA_AAG_AAT_TAA_AAC_CTA_GAA_AGG_AAA_GTG_ATA_CCC_CTA_CCA_AAA_AAA_CTC_GTA_TAA	9% ±4.39
	250390611	AAA_GAT_AGA_ATT_AAT_CAT_AAT_AAA_AAG_CCT_CTA_AAA_AAA_AGA_CTA_AAA_TAG_CTG_AAA_CTA_AGG_AAA_TAA_AAG_TAA_GTA_CAA_CTG_ATC_CAG_ACA_GTG	24% ±5.01

Table S3 Known genes with phase variation in bacteria (van der Woude MW and Bäumler AJ, 2004)

<table>
<tr><th>Gene Name</th><th>GenBank ID</th><th>Organism</th></tr>
<tr><td>LgtC</td><td>gi|15991373|gb|AAL12839.1|AF355193_3</td><td>Neisseria meningitidis</td></tr>
<tr><td>DNA methylase</td><td>gi|221232312|ref|YP_002511465.1|</td><td>Streptococcus pneumoniae</td></tr>
<tr><td>HifB</td><td>gi|535166|emb|CAA83901.1|</td><td>Haemophilus influenzae</td></tr>
<tr><td>bvgS</td><td>gi|33592925|ref|NP_880569.1|</td><td>Bordetella pertussis Tohama I</td></tr>
<tr><td>FlhA</td><td>gi|57021029|gb|EAL57693.1|</td><td>Campylobacter coli RM2228</td></tr>
<tr><td>pgtA</td><td>gi|20219024|gb|AAM15779.1|AF485419_1</td><td>Neisseria gonorrhoeae</td></tr>
<tr><td>LgtA</td><td>gi|21069205|gb|AAM33875.1|AF470665_1</td><td>Neisseria meningitidis</td></tr>
<tr><td>FliP</td><td>gi|9663108|emb|CAC01117.1|</td><td>Helicobacter pylori</td></tr>
<tr><td>pilE</td><td>gi|2073462|emb|CAA73472.1|</td><td>Neisseria meningitidis</td></tr>
<tr><td>PilS</td><td>gi|254805897|ref|YP_003084118.1|</td><td>Neisseria meningitidis alpha14</td></tr>
<tr><td>SpxB</td><td>gi|32744845|gb|AAP87106.1|</td><td>Streptococcus pneumoniae D39</td></tr>
<tr><td>Mod</td><td>gi|339905947|gb|AEK25046.1|</td><td>Helicobacter pylori</td></tr>
<tr><td>lgtD</td><td>gi|1857121|gb|AAB48386.1|</td><td>Neisseria meningitidis</td></tr>
<tr><td>wlaN</td><td>gi|218562753|ref|YP_002344532.1|</td><td>Campylobacter jejuni</td></tr>
<tr><td>Mod</td><td>gi|126508369|gb|ABO15370.1|</td><td>Haemophilus influenzae</td></tr>
<tr><td>Ag43</td><td>gi|49176177|ref|YP_026164.1|</td><td>Escherichia coli str. K-12</td></tr>
<tr><td>FetA</td><td>gi|194100042|ref|YP_002003182.1|</td><td>Neisseria gonorrhoeae</td></tr>
<tr><td>fim3</td><td>gi|33592658|ref|NP_880302.1|</td><td>Bordetella pertussis Tohama I</td></tr>
<tr><td>futA</td><td>gi|254779629|ref|YP_003057735.1|</td><td>Helicobacter pylori B38</td></tr>
<tr><td>futB</td><td>gi|254779362|ref|YP_003057467.1|</td><td>Helicobacter pylori B38</td></tr>
<tr><td>futC</td><td>gi|134142865|gb|ABO61750.1|</td><td>Helicobacter pylori</td></tr>
<tr><td>HifA</td><td>gi|535170|emb|CAA83905.1|</td><td>Haemophilus influenzae</td></tr>
<tr><td>hmbR</td><td>gi|209363433|gb|ACI43989.1|</td><td>Neisseria meningitidis</td></tr>
<tr><td>hpuAB</td><td>gi|9695428|gb|AAC44893.2|</td><td>Neisseria meningitidis</td></tr>
<tr><td>lic2A</td><td>gi|305380|gb|AAA65534.1|</td><td>Haemophilus influenzae</td></tr>
<tr><td>Lic3A</td><td>gi|309750227|gb|ADO80211.1|</td><td>Haemophilus influenzae R2866</td></tr>
<tr><td>licA</td><td>gi|126275|sp|P14181.1|LICA2_HAEIF</td><td>Haemophilus influenzae</td></tr>
<tr><td>LicA</td><td>gi|313667743|ref|YP_004048027.1|</td><td>Neisseria lactamica 020-06</td></tr>
<tr><td>maf1</td><td>gi|284926542|gb|ADC28894.1|</td><td>Campylobacter jejuni</td></tr>
<tr><td>opa</td><td>gi|110559845|gb|ABG76159.1|</td><td>Neisseria meningitidis</td></tr>
<tr><td>opc</td><td>gi|150271|gb|AAA25461.1|</td><td>Neisseria meningitidis</td></tr>
<tr><td>PilC</td><td>gi|1246818|emb|CAA90909.1|</td><td>Neisseria meningitidis</td></tr>
<tr><td>pldA</td><td>gi|298736179|ref|YP_003728705.1|</td><td>Helicobacter pylori B8</td></tr>
<tr><td>PorA</td><td>gi|7839495|gb|AAF70297.1|AF255001_1</td><td>Neisseria meningitidis</td></tr>
<tr><td>SclB</td><td>gi|13235596|emb|CAC33780.1|</td><td>Streptococcus pyogenes</td></tr>
<tr><td>SiaA</td><td>gi|520733|gb|AAA20475.1|</td><td>Neisseria meningitidis</td></tr>
<tr><td>SiaD</td><td>gi|159154704|gb|ABW93688.1|</td><td>Neisseria meningitidis</td></tr>
<tr><td>UspA1</td><td>gi|26284390|gb|AAN84895.1|AF352398_1</td><td>Moraxella catarrhalis</td></tr>
</table>

Table S4 GeneTack clusters with members homologous to known genes with phase variation. **Query gene name** – the name of a gene with known phase variation; **# hits (clustered)** – total number of fs-proteins with hits to query in BLASTp search (how many of them belong to fs-clusters); **Main GeneTack cluster** – the name of fs-cluster with the largest number of BLASTp hits; **Size (# hits)** – size of the main cluster (number of fs-proteins with hits to the query in BLASTp search).

Query gene name (Organism)	Function	# hits (clustered)	Main GeneTack cluster	Size (# hits)
DNA methylase (Streptococcus pneumoniae)	DNA modification	106 (82)	DNA methylase	59 (59)
Mod (Helicobacter pylori)	DNA modification	38 (27)	Methyltransferase	6 (6)
FlhA (Campylobacter coli)	Flagella	143 (130)	Bac_export_2, FHIPEP	72 (72)
FliP (Helicobacter pylori)	Flagella	134 (116)	FliO, FliP	48 (48)
SpxB (Streptococcus pneumoniae)	Metabolism	170 (134)	TPP_enzyme	56 (56)
HifB (Haemophilus influenzae)	Pilus	67 (53)	Pili_assembly	21 (21)
pilE (Neisseria meningitidis)	Pilus	13 (7)	Pilin	5 (5)
PilS (Neisseria meningitidis)	Pilus	13 (7)	Pilin	5 (5)
LgtA (Neisseria meningitidis)	Transferase	100 (34)	Glycos_transf_2	8 (7)
LgtC (Neisseria meningitidis)	Transferase	30 (25)	Glyco_transf_8	17 (17)
lgtD (Neisseria meningitidis)	Transferase	31 (10)	Glycos_transf_2	5 (5)
wlaN (Campylobacter jejuni)	Transferase	96 (34)	Glycos_transf_2	5 (5)
pgtA (Neisseria gonorrhoeae)	Transferase	36 (18)	Glycos_transf_1, Epimerase	8 (8)
bvgS (Bordetella pertussis)	Regulation	250 (132)	HATPase_c, HisKA, Response_reg	83 (59)

Frequencies of frameshift prone sequences in genes with high expression levels

Frequencies of occurrence of frameshift prone hexamer/heptamers (patterns with and without code frame) were determined in protein coding genes of three genomes: *E. coli*, *H. influenzae* and *V. cholerae*. We attempted to determine if the frequencies of frameshift prone sequences in sets of highly expressed genes are lower than in other genes.

For each gene set a table of all observed hexamers/heptamers counts was prepared and sorted by the counts (with most frequent on the top). We compiled the tables below from the original tables with counts by extracting rows corresponding to frameshift prone patterns. The sets of highly expressed genes were taken from Karlin, S., et al., J Bacteriol, 2001. 183(17): p. 5025-40.

Rank – row number for the pattern in the original table (more frequent sequences have higher rank); Gene count – total number of genes containing the pattern; Total count – total number of times the pattern was observed in the dataset; Log10(freq) – calculated for the pattern frequency in the given set of genes, defined as the_pattern_count/SUM_of_all_pattern_counts.

Table S5 * 49152 = $3*4^7$.

	High exp *E. coli* genes (325)	Other *E. coli* genes (3964)	High exp *H.inf* genes (158)	Other *H. inf* genes (1716)	High exp *V. cholerae* genes (173)	Other *V. cholerae* genes (2736)
**A_AAA_AAG**	49152*	17130	49152*	14519	49152*	14618
**A_AAA_AAC**	17427	10641	5226	4978	16305	10410
**TTA_AAA_A**	20567	4854	1168	229	49152*	3631
**AAA_AAA_T**	4180	2247	291	112	11462	3416
**AAAAAAA**	6477	1056	95	63	7206	887
